# Persistent humid climate favored the Qin and Western Han Dynasties in China around 2,200 y ago

**DOI:** 10.1073/pnas.2415294121

**Published:** 2024-12-23

**Authors:** Chun Qin, Bao Yang, Achim Bräuning, Fredrik Charpentier Ljungqvist, Timothy J. Osborn, Vladimir Shishov, Minhui He, Shuyuan Kang, Lea Schneider, Jan Esper, Ulf Büntgen, Jussi Grießinger, Danqing Huang, Peng Zhang, Stefanie Talento, Elena Xoplaki, Jürg Luterbacher, Nils Chr. Stenseth

**Affiliations:** ^a^Key Laboratory of Ecological Safety and Sustainable Development in Arid Lands, Northwest Institute of Eco-Environment and Resources, Chinese Academy of Sciences, Lanzhou 730000, China; ^b^School of Geography and Ocean Science, Nanjing University, Nanjing 210023, China; ^c^Institute of Geography, Friedrich-Alexander-University Erlangen-Nürnberg, Erlangen 91058, Germany; ^d^Department of History, Stockholm University, Stockholm 106 91, Sweden; ^e^Bolin Centre for Climate Research, Stockholm University, Stockholm 106 91, Sweden; ^f^Swedish Collegium for Advanced Study, Uppsala 752 38, Sweden; ^g^Climatic Research Unit, School of Environmental Sciences, University of East Anglia, Norwich NR4 7TJ, United Kingdom; ^h^Mathematical Methods and Information Technology Department, Siberian Federal University, Krasnoyarsk 660075, Russia; ^i^Department of Geography, Justus Liebig University of Giessen, Giessen 35930, Germany; ^j^Department of Geography, Johannes Gutenberg University, Mainz 55099, Germany; ^k^Global Change Research Institute (CzechGlobe), Czech Academy of Sciences, Brno 603 00, Czech Republic; ^l^Department of Geography, University of Cambridge, Cambridge CB2 3EN, United Kingdom; ^m^Department of Geography, Faculty of Science, Masaryk University, Brno 611 37, Czech Republic; ^n^Swiss Federal Research Institute for Forest, Snow and Landscape Research, Birmensdorf 8903, Switzerland; ^o^Department of Environmental and Biodiversity, University of Salzburg, Salzburg 5020, Austria; ^p^School of Atmospheric Sciences, Nanjing University, Nanjing 210023, China; ^q^Centre for Ecological and Evolutionary Synthesis, Department of Biosciences, University of Oslo, Oslo 0316, Norway

**Keywords:** climate variability, ancient dynasties, climate reconstruction, climate impacts, Asian summer monsoon

## Abstract

The Qin and Western Han dynasties (221 BCE to 24 CE) represent an era of societal prosperity in China. However, due to a lack of high-resolution paleoclimate records it is still unclear whether the agricultural boost documented for this period was associated with more favorable climatic conditions. Here, multiparameter analysis of annually resolved tree-ring records and process-based physiological modeling provide evidence of stable and consistently humid climatic conditions during 270 to 77 BCE in northern China. Precipitation in the Asian summer monsoon region during the Qin–Western Han Dynasties was ~18 to 34% higher compared to present-day conditions. In shifting agricultural and pastoral boundaries ~60 to 100 km northwestward, possibility up to 200 km at times, persistently wetter conditions arguably increased food production, contributing to the socioeconomic prosperity around 2,200 y ago. A gradual wetting trend in the western part of arid northwestern China since the 1980s resembles the historical climate analogue, suggesting that similar benefits for regional environmental and agricultural systems may reoccur under current climate change, at least in the near term.

## Significance

It has remained unclear whether the socioeconomic prosperity of the Qin–Western Han dynasties (221 BCE to 24 CE) in China was linked to climate conditions favoring agricultural productivity. We demonstrate, using an annually resolved precipitation reconstruction for northern China, that stable and consistently humid climatic conditions prevailed in northern China during this period. The annual total precipitation during the Qin–Western Han dynasties was ~18 to 34% higher than during 1951 to 2015 CE. By comparing the locations of ancient cities with the northwestern boundary of modern rain-fed agriculture, we provide strong evidence that climate change had a major impact on human society during the Qin–Western Han dynasties by enabling a northwestward extension of agricultural activity.

Under recent global warming, precipitation patterns have changed across most of the world (1, 2), and more pronounced changes are projected in the coming decades (3). However, the amplitude (and, in some regions, even the direction) of projected changes remain uncertain (4). While most global climate models project increased precipitation at the northern fringe of the Asian summer monsoon (NASM), there is weak confidence in the magnitude of the change due to discrepancies among individual models and between model ensembles (CMIP5 versus CMIP6) (5), and because of a persistent northward bias in the position of the boundary of the NASM in most CMIP6 models (6). Other approaches are therefore needed to complement climate model projections and provide a more complete understanding of hydroclimate change in the NASM region. One such approach is to reconstruct and assess past hydroclimate conditions during historical warm climatic periods using high-resolution proxy data (7, 8).

**Fig. 1.**
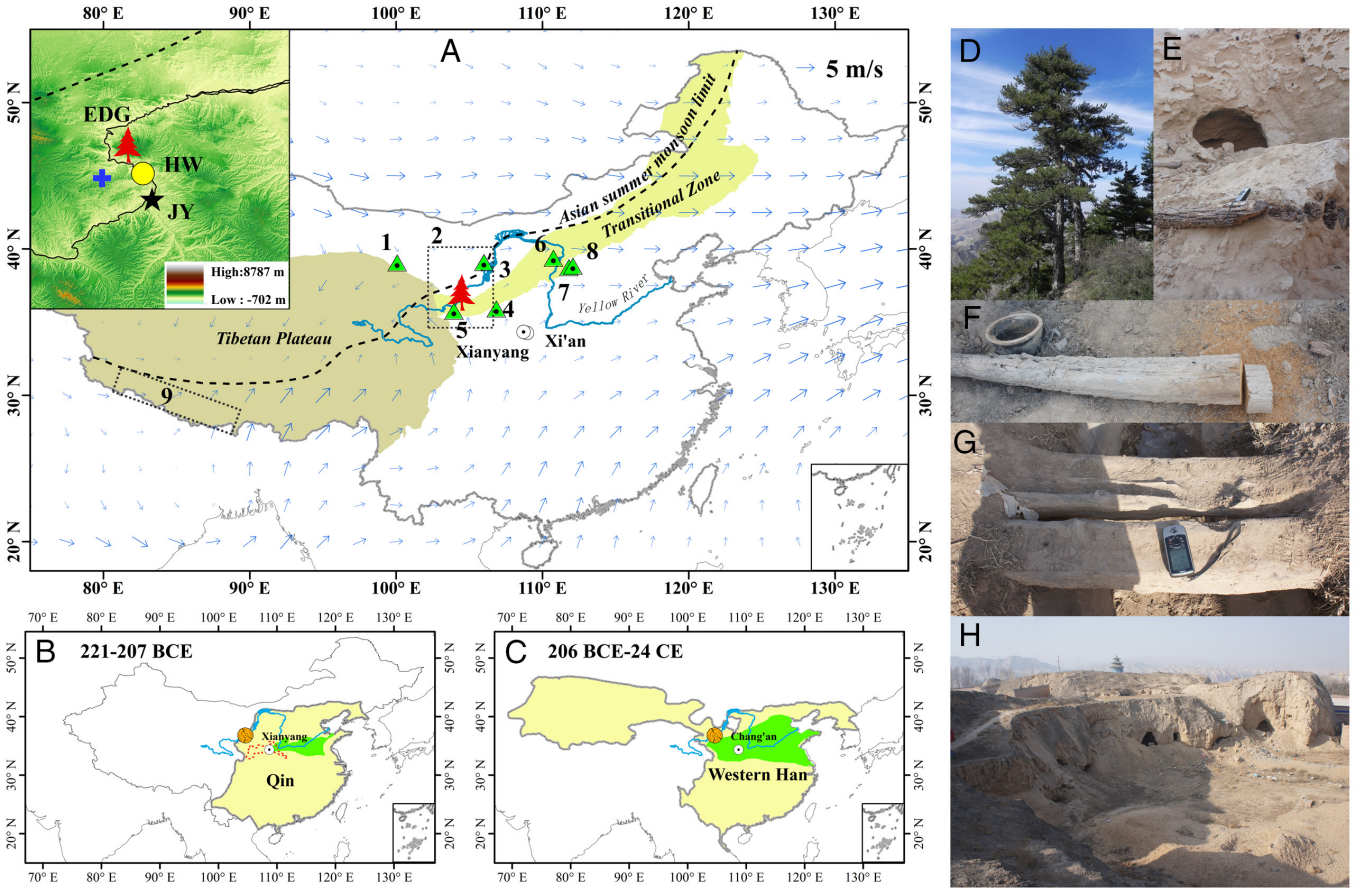
The study region and sample materials. Location of living pine (the red tree symbol) sampling site, the capitals of the Qin Dynasty (Xianyang) and Western Han Dynasty (Xi’an, named Chang’an at that time) (*A*) Other proxy sites are numbered 1 to 9, with metadata provided in *SI Appendix*, Table S5. The black dashed line marks the modern Asian summer monsoon (ASM) limit (modified from ref. 12); the arrows indicate the 700 hPa mean summer wind velocity (1951 to 2018 CE mean of NCEP/NCAR reanalysis); the light green region shows the farming-pastoral transition area with 1951 to 2018 annual precipitation between 300 and 450 mm. The inset map shows the sampling site of living Chinese pine in Jingyuan county (EDG, red tree), the archeological wood (AW) sampling site-Huangwan tomb (HW, yellow dot), the Jingyuan meteorological station (JY, black star), and the scPDSI data grid points (blue plus signs). Maps of the territorial (yellow) and approximate farmland (green, modified from ref. 13) extents during the Qin (*B*) and Western Han (*C*) dynasties. The red dashed line in (*B*) outlines the territory of the Qin state (roughly 441 to 389 BCE). Photo (*D*) shows living pine at EDG; the status of the AW collected in this study is illustrated by photos (*E* and *F*) exposed and weathered or photo (*G*) used as a country road; photo (*H*) shows the environment surrounding the ancient tomb and the adjacent human residences.

The Qin (221 to 207 BCE) and the following Western Han (206 BCE to 24 CE) dynasties (hereafter referred to as the Qin–Western Han dynasties, 221 BCE to 24 CE) experienced a climate that, at least during certain intervals, was as warm as or even slightly warmer than that of the 1981 to 2010 CE period (9, 10). These dynasties were characterized by economic well-being and a generally prosperous society, providing a backdrop of favorable conditions for the first great “reunification” of China (Fig. 1). In many ways this period in Chinese history has parallels to the early expansion phase of the Roman Empire in the Mediterranean region (11). However, a lack of high-resolution proxy records has hitherto made it difficult to constrain the historic hydroclimate variability of northern China, leaving our understanding of the link between climate and societal prosperity during the Qin–Western Han dynasties largely incomplete.

Regions at the northern fringe of the ASM (highlighted in Fig. 1*A*) are particularly sensitive to climate change (14–16) (*SI Appendix*, Fig. S1). Variations in regional water availability have historically strongly affected human well-being and social development, e.g., the famine of 1876 to 78 CE contributed to ~23 million deaths in northern China (17). Currently available hydroclimate reconstructions derived from tree-rings disagree on the overall wetness or dryness of conditions during the Qin–Western Han dynasties (18–21). Furthermore, these existing records originate from the highlands of western China, and there is a pressing need for a reliable record from central China, closer to the capital at that time and more conducive to understanding the relationship between hydroclimate change and social progress. However, the lower temporal resolution and age uncertainties of existing central China records (22–24) make it difficult to draw convincing conclusions.

In this study, we use multiple parameters from absolutely dated tree-ring records (*SI Appendix*, Table S1) from central China to better assess the extent and duration of wet and dry conditions. These provide a sounder basis for discussing potential climate impacts on societal development and economic well-being during the Qin–Western Han dynasties. Furthermore, we consider reconstructed climate conditions from the past as analogues with which to evaluate possible regional effects of future climate change.

## Results

### More Favorable Tree Growth Conditions During the Qin–Western Han Dynasties.

**Fig. 2.**
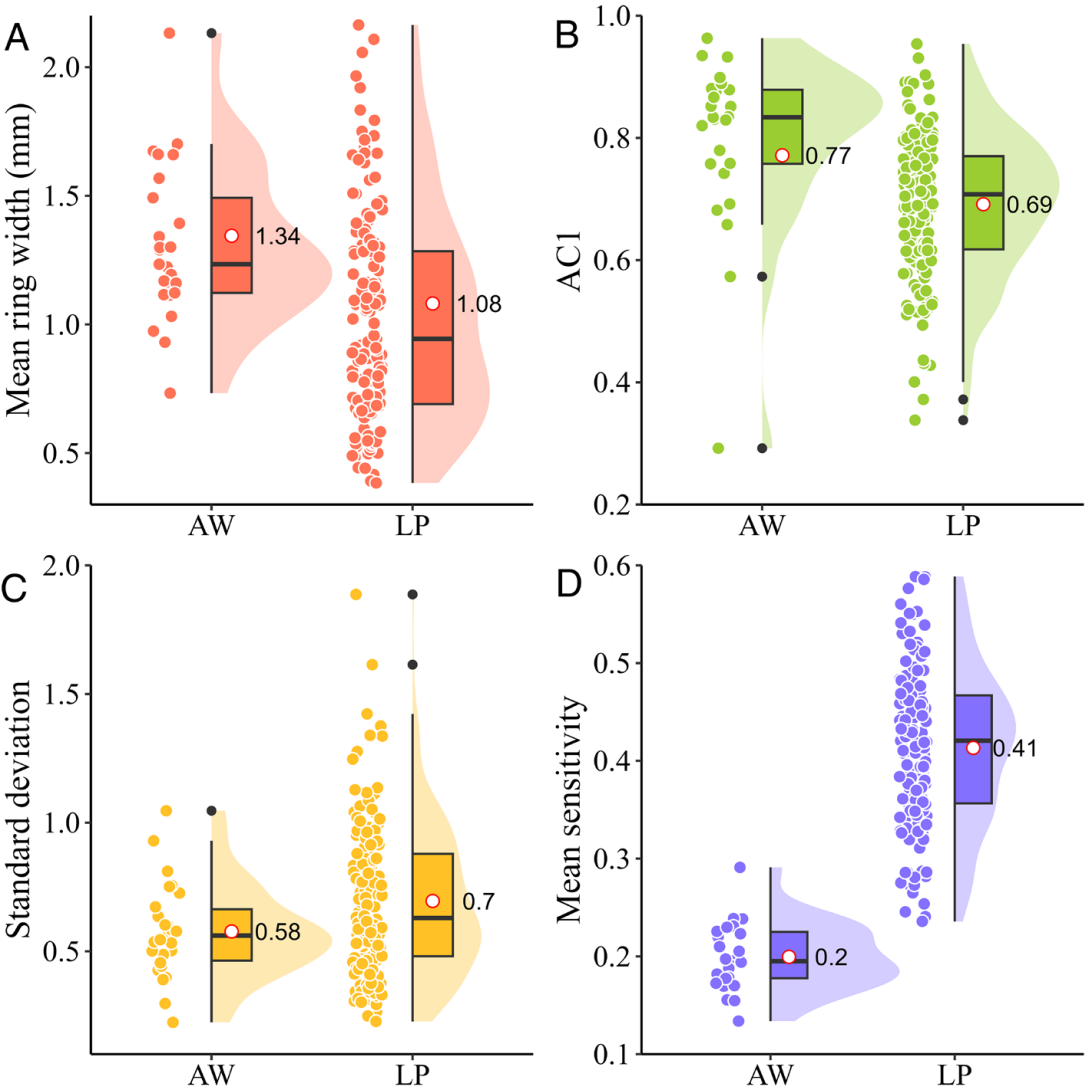
Increased growth rate and reduced growth variability of AW compared with modern living pines. Box plots, distribution curves, and individual tree statistics (overlapping data points) for mean ring width (*A*), first-order autocorrelation coefficient (AC1, *B*), SD (*C*), and mean sensitivity (*D*) of the AW and living Chinese pine (LP) data calculated over the corresponding credible periods (270 to 77 BCE and 1751 to 2015 CE). Box plots show the median (center line), mean (hollow red dot and labels), and upper and lower quartiles (box boundaries). The vertical lines extend 1.5 times the interquartile range beyond the box boundaries.

**Fig. 3.**
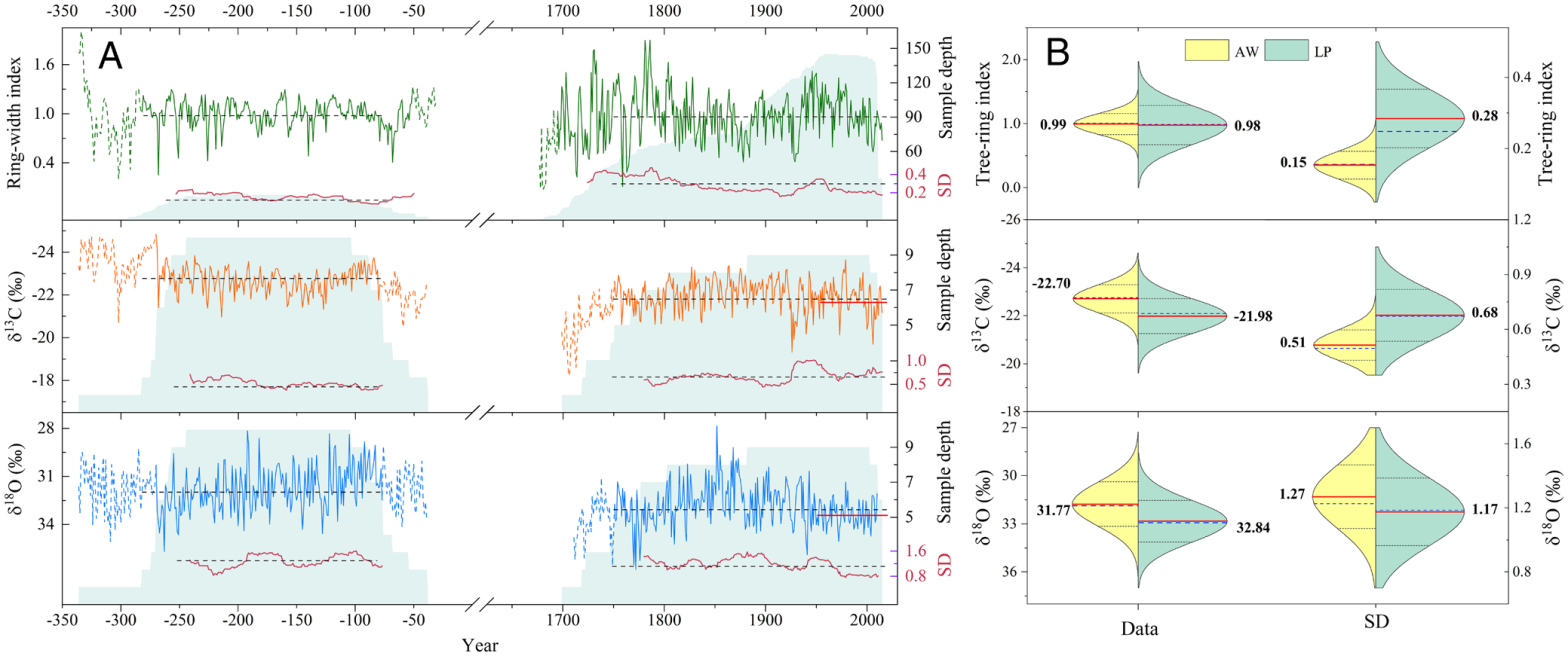
Tree-ring chronologies illustrate differences in mean isotope ratios and variability between the archeological and living pine measurements. (*A*) Ring-width (TRW; standardized which removes differences in mean growth rate), cellulose δ^18^O, and δ^13^C chronologies shown together with sample depth (blue shading), and running SD (SD, red lines) of each tree-ring variable. Timeseries are shown as solid lines during their credible periods (EPS > 0.85 and replication of ≥5 trees or wood blocks) and as dashed lines when they are less reliable. Sample depth (blue shading, right-hand scale) shows the number of trees; running SD (right-hand scale) is computed using a 30-y running window for each credible period; means over 270 to 77 BCE and 1751 to 2015 CE are shown as horizontal dashed lines, and over 1951 to 2015 CE by a horizontal red line. (*B*) Statistical distribution of each record and their running SD. Split violin plots are marked with means (red lines and labels), medians (blue dashed lines), and ±1 SD (black dotted lines).

Compared with the recent 1951 to 2015 CE and 1677 to 2015 CE periods, the newly developed ring-width series during the Qin–Western Han dynasties (270 to 77 BCE) exhibit significantly wider mean tree-ring width (TRW), significantly higher first-order autocorrelation (AC1), and significantly lower SD (Fig. 2 *A*–*C* and *SI Appendix*, Table S2). The higher AC1 and lower SD combine to yield a much-reduced mean sensitivity (Fig. 2*D*), indicating less extreme environmental conditions during the tree growth (25). Further analysis reveals that wider ring widths during the Qin–Western Han period (+22 to 30%) are independent of tree age (*SI Appendix*, Fig. S5 and Table S3). The mean tree-ring δ^13^C and δ^18^O values during the Qin–Western Han dynasties were lower than the mean of 1751 to 2015 (by 0.72‰ for δ^13^C and 1.06‰ for δ^18^O) and even lower than the mean of the 1951 to 2015 instrumental period (by 0.85‰ for δ^13^C and 1.57‰ for δ^18^O, respectively) (Fig. 3 and *SI Appendix*, Table S4). Such depleted δ^13^C and δ^18^O values are the result of wetter-than-present conditions.

The TRW and δ^13^C 30-y running SD (Fig. 3*A*), as well as distributions of both metrics (Fig. 3*B*) show clearly reduced temporal variability for 270 to 77 BCE period compared with the recent tree-ring data, indicating that the climate during the Qin–Western Han dynasties was more stable compared to modern times (Fig. 3). Reduced variability is not apparent in the δ^18^O chronology, but this proxy is less strongly linked to local hydroclimate variability. Taken together, these statistics suggest that the climate during the Qin–Western Han dynasties was consistently stable and humid, with lower year-to-year hydroclimatic variability, and thus was more favorable for tree growth compared to recent periods (1751 to 2015 or 1951 to 2015 CE).

### Modern Observations Link Tree-Ring Proxies to Hydroclimate.

The TRW and δ^13^C series are strongly influenced by drought, relative humidity, and precipitation during the calibration period 1951 to 2015 CE (*Materials and Methods*, *SI Appendix*, Figs. S6–S7), and demonstrate significant spatial correlation across the northern fringe of the ASM (*SI Appendix*, Fig. S9 and S10). Correlations between tree-ring δ^18^O and local hydroclimate indicators are weaker, but there is good agreement during recent centuries (*SI Appendix*, Tables S6 and S9 and, Figs. S15 and S16) between the δ^18^O timeseries and other tree-ring δ^18^O records from monsoonal Asia that have been interpreted to represent monsoon intensity (Fig. 1*A* and *SI Appendix*, Table S5 for detailed information on each proxy). This good agreement in both high- and low-frequency domains suggests that our δ^18^O record is a useful indicator of regional variations in ASM intensity (26).

### Quantitative Precipitation Reconstruction and Verification.

We reconstructed precipitation during the Qin–Western Han dynasties using a linear regression analysis between tree-ring δ^13^C and annual precipitation (previous August to current July, P8-C7) calibrated for the 1951 to 2015 CE period. This annual precipitation reconstruction explains 32% (F = 29.30 > F0.01 (1, 27), *P* <0.01) of the variance in the instrumental precipitation record (*SI Appendix*, Tables S7 and S8 and Fig. S11), and the regression residuals passed the tests for autocorrelation influences (*SI Appendix*, Fig. S12 and Table S8). Our reconstruction represents precipitation variations across the northern fringe of the ASM as well as drought occurrence in northern China on annual to decadal timescales (*SI Appendix*, Figs. S9, S10*B*, S15, and S16).

Another calibration was carried out based on the regional precipitation from 39 stations in the NASM region. The strong correlation (*r* = 0.64, n = 64, *P* < 0.001, *SI Appendix*, Fig. S13) indicates that our reconstruction is more reliable on a broader spatial domain in northern China. The fact that our δ^13^C chronologies can represent the precipitation at NASM is also confirmed by other regional precipitation reconstructions (*SI Appendix*, Fig. S14) (28).

Applying the regression model derived from δ^13^C data of modern, living trees to the archaeological data yields a total annual precipitation that is 16 ± 6% (~38 mm/yr, ~10 to 22%, significant at 95% confidence level) higher during the Qin–Western Han dynasties compared to that of the post-1951 calibration period in Jingyuan meteorological station at NASM. This δ^13^C-based evidence for increased precipitation in the early period is further supported by the wider mean TRW and significantly depleted δ^18^O during the Qin–Western Han dynasties (Fig. 3).

More than one third of the extreme climate events derived from our reconstruction (*SI Appendix*, Table S11) were also recorded in historical documents (*SI Appendix*, Table S11), despite these documents being subjective and discrete in time. For example, the severe drought in 158 BCE in Guanzhong area caused a series of environmental and socioeconomic impacts, such as a summer locust plague and a borer plague in August. This severe disaster prompted the government to take a series of measures, including tax reductions, lowering tributes, cutting expenditures, distributing grain for disaster relief, and buying/selling official positions (29). The scale of this drought event is unprecedented and has been recorded in many other places in China. Heavy rains, lasting from July to October in 86 BCE, were also recorded and this appears as a pluvial year in our reconstruction. This heavy rain caused the river to surge, destroying Wei Bridge, and high soil moisture for two subsequent years, though its impact on the local population lasted even longer (it was reported that the refugees had not returned by 83 BCE (29).

Tree-ring hydroclimatic reconstructions based on linear regression models (30) tend to underestimate the magnitude of climate variability, suggesting that the reconstructed ~16% higher mean precipitation during the Qin–Western Han dynasties relative to present regional climate conditions is likely to be a minimum estimate. The process-based Vaganov-Shashkin oscilloscope (VS) model provides complementary, non-linear insights into the link between TRW and past climate (31). VS simulations, using parameters (*SI Appendix*, Table S10) appropriate to this region and tree species prescribed or determined by calibration (*SI Appendix*, Fig. S17), confirm that the radial growth of trees in this region is much more sensitive to precipitation than to temperature (*SI Appendix*, Fig. S18). Based on this model, the measured ~ 22 to 30% wider mean TRW during the Qin–Western Han dynasties would have required precipitation to have been ~17 to 34% (39 to 79 mm) higher than during the post-1951 calibration period, provided that mean temperature had remained within ±0.5 °C of present-date (*SI Appendix*, Fig. S18).

**Fig. 4.**
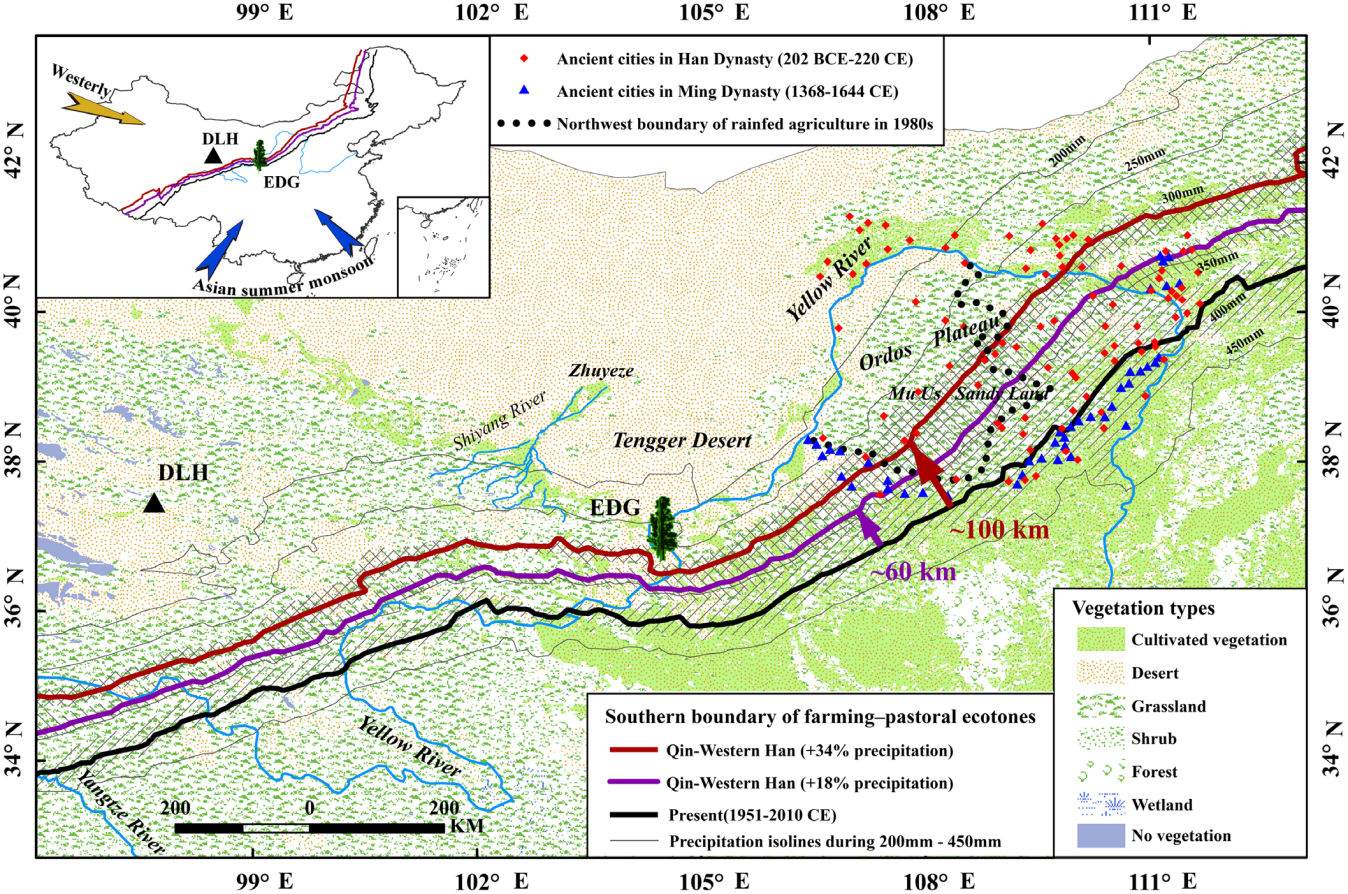
Migration of farming-pastoral ecotones during the Qin–Western Han Dynasties under different precipitation scenarios (+18 to +34% compared with current precipitation). The modern (1960 to 2018 CE) precipitation isohyets are shown faintly for 200 to 450 mm/y in steps of 50 mm/y. Present-day isohyets of 340 mm/y and 300 mm/y are shown in purple and red because increases in mean precipitation of 18% and 34% would raise these to 400 mm/y (present-day isohyet shown in black) which is considered to determine the southern boundary of the farming-pastoral transition area and almost coincides with the ASM boundary in China. The movement of these isohyets is indicated by approximate distances and compared with their modern interdecadal variability (hatching) and to the locations of ancient cities (modified from ref. 32 and interpreted as permanent settlements of agricultural populations) during the Han Dynasty (red diamonds) and the colder/drier Ming Dynasty (blue triangles). The black dotted line marks the northwestern boundary of rainfed agriculture on the Ordos Plateau modified from ref. 33's manual interpretation of Landsat imagery in 1980s. The sampling sites EDG (green tree) and DLH (our previously published 6,700-y tree-ring isotope record, black triangle) are also marked. The *Inset* indicates the general context of the sampling sites and the isohyets. *SI Appendix*, Fig. S19 gives further information.

Moist conditions during the Qin–Western Han period are also indicated by a tree-ring δ^18^O series from the Delingha (DLH) region (600 km northwest of our study site, Fig. 4), where reconstructed annual precipitation was 20% higher than during the 1951 to 2015 CE period (21). Generally moist conditions in northwestern China are also seen in lower-resolution natural proxy archives—e.g., ice cores, stalagmites, river, and lake sediments—as well as in historical documentary evidence (22–24). Thus, multiple lines of evidence are consistent with our conclusions that the Qin–Western Han dynasties were ~18 to 34% wetter than the average of the post-1951 period.

According to modern rainfall distribution, ~18 to 34% higher precipitation during the Qin–Western Han period would imply a ∼60 to 100 km northwestward migration of the ASM boundary at that time (Fig. 4). The maximum fluctuation range of the transition zone even reaches 200 km on the Ordos Plateau (Fig. 4).

## Discussion

### Impact of Climate on the Environment, Economy, and Society.

Based on our precipitation reconstruction, we conclude that the stable and consistently warm and humid climate conditions during the Qin–Western Han dynasties favored large-scale agricultural food production and promoted regional economic and demographic prosperity. Supporting evidence for a prevailing humid climate in the northern ASM region during this period comes from a large lake called “Zhuyeze” (34) in the Minqin area (Fig. 4, considered to be the source area of current sandstorms, located 300 km northwest of our study site), and from historical documentary data showing that local forest coverage was higher during the Qin–Western Han dynasties than that at present (9). Our findings about the societal impacts of past climatic extremes do not contradict the notion of a stable climate during the Qin–Western Han dynasties. An extreme event should be assessed based on a reference period that reflects society’s capacity for adaptation. The synchronization between climatic extremes and recorded societal impacts validates the accuracy of these two independent sources of data. While historical records can often be subjective and irregular over time, our reconstruction offers a consistent, quantitative benchmark that provides much-needed context for periods with limited data (35). This allows for a more objective understanding of past climate–society interactions.

Agricultural subsistence strategies aimed to supply sufficient local and regional food across NASM. Historical records reveal that the rice planting area expanded northward to both the Hexi region (36) and even the Yellow River Basin (37), where rice agriculture is not commonly practiced today due to the rare fulfillment of necessary hydrothermal requirements. Historical documents also report the abundance of bamboo in the Yellow River Basin, which prefers a warm and humid environment (10, 37). “Solar terms” were the farming time regulations formulated by the ancient Chinese according to climatic conditions and crop phenology. During the Qin–Western Han dynasties, the order of solar terms was adopted as first “Guyu” (Grain Rain) and then “Qingmin” (Pure Brightness) (10, 37), indicating that spring sowing occurred 15 d earlier than it does today. According to the ancient book “Liji·Yue Ling”, this change in the order of solar terms is due to changes in climate conditions, and earlier spring sowing indicates warmer and humid climatic conditions (37). Importantly, the “tuntian” policy that stipulated soldiers must plant crops where they were stationed or recruit farmers to do so for them could prevail in the region during the period (38). The success of these measures in ensuring sufficient agricultural activity cannot be explained solely by the fact that it was an administrative order; it is more likely evidence of adaptation to the favorable regional climate conditions of the time. A network analysis of more than 10,000 historical documentary records has revealed that precipitation was the single-most important factor for harvest success or crop failure during historical times in China (39). This emphasizes the importance of more humid climatic conditions for rain-fed agriculture at the northern fringe of the ASM region.

There was a persistent trend of human migration to the northwest during the Qin–Western Han Dynasties. Archaeological evidence showed that ancient cities in the Hexi Corridor were more numerous and more widely distributed during the Han dynasty than any other historical period during the past 5,000 y (40). Furthermore, a similar pattern of an increasing number of ancient cities extended to the Mu Us Desert (300 km northeast, Fig. 4) and surrounding areas along NASM (32, 41). The ancient cities on the Ordos Plateau were permanent settlements supported by local rainfed agriculture. An increase of at least 20% of present-day annual precipitation in the Mu Us Desert (250 to 350 mm/y, Fig. 4) would have provided favorable conditions (300 to 420 mm/y) for the development of agricultural production if it remained stable and persistent. Insufficient precipitation in a drought-limited growth environment will not meet the basic conditions for premodern rainfed agriculture, regardless of the agricultural policies in place. Therefore, the northwestern boundary of ancient cities at NASM can also be regarded as the northwestern boundary of rainfed agriculture. As shown in Fig. 4, ancient cities (42) during the Ming dynasty (a colder and drier period compared to the present) shifted ~75 km southeast of the boundary of present rainfed agriculture, while ancient cities during Han Dynasty extended ~40 to 100 km beyond the northwestern boundary of present-day rainfed agriculture. Therefore, it is reasonable to infer that the stable and humid favorable climatic conditions in the rainfed agricultural area ensured abundant crop yields and the expansion of farmland to the northwest (Fig. 1 *B*–*C*) during the Qin–Western Han dynasties, providing food resources for the growing population.

Overall favorable climatic conditions for agriculture doubled the farming area and tripled the population of China during the Qin–Western Han Dynasties (9). In addition, large-scale rice cultivation began in northern China during the Qin–Western Han dynasties (9). All these changes contributed to successful and high-yielding harvests (43, 44) and a lack of famines, enabling societal development and demographic growth of the Qin–Western Han dynasties (43, 45). There is general agreement on the impacts of climate change in the history of China, with negative impacts during cold periods and positive impacts during warm periods on centennial time-scales (46, 47). Our study provides evidence that increased precipitation, and reduced precipitation variation, under warm conditions had a major positive impact on human society.

### Implications for the Future.

Favorable climatic conditions, combined with effective environmental management policies, can greatly improve the regional ecological environment. The prevailing warm and humid climate during the Qin–Western Han dynasties offers a possible analogue for the near-future climate in northern China and is therefore of significance for climate stakeholders and policy makers. Indeed, a trend toward wetter conditions since 1987 CE (which started in the western part of arid northwest China and progressed eastward in subsequent decades: *SI Appendix*, Fig. S20), combined with multiple afforestation programs in China (e.g., the Three-North Shelterbelt Development Program, the Nature Forest Conservation Program, and the Grain to Green Program), has been accompanied by increased river runoff and lake levels, and fewer sandstorms (22). Recent research shows that this warming and wetting trend in northwest China has continued (48), suggesting that near-future prospects for this region may resemble the warm and humid climate of the Qin–Western Han dynasties, with associated benefits of increased vegetation cover in northern China as recorded over 2,200 y ago. Although such climate conditions are favorable for the ecological environment in northwest China, we emphasize that a strengthening ASM is related to larger-scale atmospheric circulation anomalies, such as reduced El Niño variability (49, 50) or an intensified North Atlantic Oscillation (51), which may increase the risk of droughts in other areas (52).

## Materials and Methods

### Tree-Ring Sampling.

Chinese pine (*Pinus tabulaeformis* Carr.) is the dominant tree species in the semiarid mountainous regions of northern China. We collected wood samples from LP at breast height in the Hasi Mountains, and AW from the Huangwan tomb, which can be dated back to the Han dynasty (Fig. 1), in Jingyuan County that is 26 km apart from the LP site. The AW samples were identified as Chinese pine according to their wood anatomical characteristics (*SI Appendix*, Fig. S2). Evidence from historical documents suggests that the timber for Huangwan tomb came from the Hasi Mountains (*SI Appendix*; Fig. S21). A total of 155 LP samples were collected from elevations ranging between 2,137 and 2,456 m a.s.l to compare with the AW samples.

### Tree-Ring δ^13^C and δ^18^O Measurement.

Cellulose δ^13^C in C3 tree species is mainly controlled by fractionation associated with the ratio of intercellular (c_i_) to ambient (c_a_) air CO_2_ concentrations (53). The c_i_/c_a_ ratio depends on the stomatal conductance and the rate of photosynthesis (54), both of which are closely related to relative humidity (rH) in semiarid and arid regions. Tree-ring cellulose δ^18^O is mainly controlled by the δ^18^O of the source water (precipitation) and ^18^O enrichment of leaf water by transpiration. The rate of transpiration depends on stomatal conductance and vapor pressure deficit between leaf air and ambient air, both of which are linked to rH (55, 56). Low rH leads to an enrichment in ^18^O due to evapotranspiration and higher δ^18^O values in tree-ring cellulose, and vice versa. The dominant environmental signals in tree-ring δ^18^O are thus the stable isotope content of the precipitation (and soil moisture) and summer rH. A more complete discussion of tree-ring stable isotope fractionation processes is available elsewhere (55–58).

Tree-ring δ^13^C and δ^18^O data used in this study were measured in three different laboratories. We avoided measuring isotopes from the first 30 y of growth to prevent age-related trends (59). The first batch of samples were measured with a pooled method in the Isotope Laboratory of the Institute of Geography at University of Erlangen-Nürnberg, Germany. Nine LP and ten AW samples from high elevation (2,338 to 2,456 m a.s.l.) (*SI Appendix*, Table S1) with clear ring boundaries, no distortions, and few missing rings were selected for δ^18^O and δ^13^C measurements. The samples were divided into annual subsamples using a razor blade, and wood from the same calendar year was pooled to minimize individual tree differences and enhance common signals (60). α-cellulose was then extracted from these pooled samples following the method of Wieloch et al. (61). After freeze drying, α-cellulose (0.3 to 0.4 mg) was separately packed in silver (for δ^18^O) and tin (for δ^13^C) capsules and measured using an elemental analyzer coupled with an isotope ratio mass spectrometer (Delta V Advantage, Thermo Scientific, Bremen, Germany). Chronologies spanning 1677 to 2010 CE for LP and 336 to 31 BCE for AW were produced and used separately to quantitatively reconstruct past and recent precipitation variations.

Another nine independent cores from living trees were measured individually at Swansea University, United Kingdom (62). The samples, collected from elevations between 2,188 and 2,415 m a.s.l., were cut into thin slivers for each ring using a scalpel before α-cellulose extraction (63). Then, 0.30 to 0.35 mg of dry α-cellulose was weighed, and placed into silver capsules for pyrolysis into CO, and isotope ratios were measured using a Flash HT elemental analyzer coupled with a Delta V IRMS. Both carbon and oxygen isotope ratios were measured simultaneously (for details, see refs. 62, 64, and 65).

The δ^13^C data from nine Chinese pines, covering 1750 to 2015 CE, were used for altitude sensitivity analysis (see *SI Appendix* for details). To maintain consistent chronology length, we extended our pooled δ^13^C record from 2010 to 2015 CE by measuring pooled rings from six new tree cores in the State Key Laboratory of Cryospheric Sciences, Chinese Academy of Sciences. All samples were collected from high elevations (2,389 to 2,412 m a.s.l.), with measurements spanning 1993 to 2015 CE.

Isotopic compositions are expressed as δ [‰] = (R_sample_/R_standard_ − 1) × 1,000‰, where R_sample_ and R_standard_ represent the ^18^O/^16^O or ^13^C/^12^C ratios of the sample and standard, respectively. The standard for ^18^O/^16^O was the Vienna Standard Mean Oceanic Water (VSMOW), and for ^13^C/^12^C was the Vienna Pee Dee Belemnite (VPDB). The SD for repeated analysis was better than 0.09‰ for δ^13^C_VPDB_ and 0.25‰ for δ^18^O_VSMOW_ in the Isotope Laboratory of the Institute of Geography (University of Erlangen-Nürnberg, Germany), 0.1‰ and 0.28‰ in the State Key Laboratory of Cryospheric Sciences (Chinese Academy of Sciences, China), and 0.1‰ and 0.3‰ in Swansea University (UK) laboratory, respectively.

### Chronology Construction and Climate Sensitivity Analysis.

TRW was measured with a 0.01 mm resolution, and cross-dated using standard dendrochronological procedures (*SI Appendix*). Three independent lines of evidence (the historical context, cross-dating with a distant reference chronology and radiocarbon dating) were used to date the AW samples (see *SI Appendix* for details). The program ARSTAN was used to construct ring-width chronologies (27). Prior to detrending, a data-adaptive power transformation, based on the local mean and SD, was used to stabilize the variance of each series. Modified negative exponential curves of any slope were used to remove age-related biological trends, and ring-width chronologies were obtained by calculating residuals between raw measurements and fitted values. Finally, two standard ring-width chronologies (TRW) were established: 1677 to 2015 CE from 155 LP samples, and 336 to 31 BCE from 26 AW samples (*SI Appendix*, Fig. S3). The δ^13^C and δ^18^O chronologies were established for the two periods using the pooled method. The δ^13^C series were corrected following the procedures described in ref. 66 to eliminate the effects of changing CO_2_ concentration during the most recent period. We grouped the corrected individual δ^13^C series by tree altitude into low-altitude and high-altitude groups. Then, we calculated the arithmetic mean of δ^13^C series for each group, and compared them to each other.

Sample depth is determined by the number of analyzed trees. An expressed population signal (67) (EPS) ≥ 0.85 and a sample depth ≥ 5 trees or wood blocks were used as criteria to define credible periods for the standardized TRW, δ^18^O, and δ^13^C chronologies.

Correlation and response function analysis of the TRW, δ^13^C, and δ^18^O series with monthly climatic variables from Jingyuan meteorological station were conducted over the period 1951 to 2015 (*SI Appendix*, Figs. S6–S8, n = 65). Correlations with monthly climate data were analyzed using the software DendroClim2002 (68) to quantify the climate sensitivity of the chronologies (see *SI Appendix* for details).

### Precipitation Reconstruction.

We used a univariate linear regression model to establish the relationship between the δ^13^C chronology and annual total precipitation (from previous August to current July, P8–C7) from Jingyuan meteorological station during 1951 to 2015 CE. This model was then used to reconstruct past precipitation variability using the δ^13^C chronology from the Qin–Western Han Dynasties. The established regression model is

[1] $${\text{PRE}}_{p8-c7}=-44.48\;\times \;\delta^{13}C\;-\;740.4\;(R^{2}=0.32).$$

Detailed information is listed in *SI Appendix*, Tables S7 and S8.

Inferring past climate and environmental conditions using discontinuous proxy records based on the assumption of uniformitarianism was routinely applied in paleoclimate domain (69–72).

### Pointer Year and Extreme Climate Events.

We identified pointer years using the δ^13^C-based precipitation reconstruction. A “pointer year” is when tree-ring data shows a significant deviation from the mean growth of the analysis period (73). We defined pointer years as those with unusually low or high annual precipitation exceeding ±1.5 SD. Years were identified as extreme pluvials or extreme droughts if the reconstructed precipitation was greater than the mean+1.5 SD or less than the mean–1.5 SD, respectively.

### The VS Model.

We used the VS model to simulate the integral tree-ring growth rate Gr(t), and to test the relative contributions of temperature and precipitation to changes in radial growth of trees in the study region. The VS model assumes that climatic influences are nonlinearly associated with tree-ring growth through controls on cell formation processes in developing wood (74). TRW series were simulated by comparing daily temperature and soil moisture budgets to growth functions using the most limiting factor (75). Tree-ring growth is described by the following equation:

[2] Gr(t)=GrE(t)×min[GrT(t),GrW(t)].

where GrE(t), GrT(t), and GrW(t) represent the partial growth rates, calculated independently based on solar irradiation, temperature, and soil moisture content (73). The parameter values for the VS model were calibrated (*SI Appendix*, Fig. S17) and selected specifically for this region and tree species (*SI Appendix*, Table S10).

Daily precipitation and temperature records from the Jingyuan meteorological station were used as input data to assess the response of wood formation to various climate change scenarios. This was done by progressively adjusted the climatic factors in the model, with daily temperature modified in 0.5 °C increments (ranging from −1 to 3 °C) and precipitation altered by 10% increments (ranging from −10 to 60%).

## Supplementary Material

Appendix 01 (PDF)

## Data Availability

Data are available from on the WDC-Paleo/ITRDB platform (76–78).

## References

[1] J. Sheffield, E. F. Wood, M. L. Roderick, Little change in global drought over the past 60 years. Nature **491**, 435–438 (2012). 23151587 10.1038/nature11575

[2] A. E. Putnam, W. S. Broecker, Human-induced changes in the distribution of rainfall. Sci. Adv. **3**, e1600871 (2017). 28580418 10.1126/sciadv.1600871 PMC5451196

[3] V. Masson-Delmotte , “Climate change 2021: The Physical Science Basis” in Contribution of Working Group I to the Sixth Assessment Report of the Intergovernmental Panel on Climate Change (2021), **vol. 2**.

[4] N. Nasrollahi , How well do CMIP5 climate simulations replicate historical trends and patterns of meteorological droughts? Water Resour. Res. **51**, 2847–2864 (2015).

[5] J. Piao, W. Chen, S. Chen, H. Gong, L. Wang, Mean states and future projections of precipitation over the monsoon transitional zone in China in CMIP5 and CMIP6 models. Clim. Change **169**, 35 (2021).

[6] J. Piao , How well do CMIP6 models simulate the climatological northern boundary of the East Asian summer monsoon? Global Planet. Change **221**, 104034 (2023).

[7] PAGES Hydro2k Consortium, Comparing proxy and model estimates of hydroclimate variability and change over the Common Era. Clim. Past **13**, 1851–1900 (2017).

[8] F. C. Ljungqvist , Northern Hemisphere hydroclimate variability over the past twelve centuries. Nature **532**, 94 (2016). 27078569 10.1038/nature17418

[9] Q. S. Ge, Climate Change in Chinese Dynasties (Science Press, Beijing, 2011).

[10] K. Z. Chu, A preliminary study on the climatic fluctuations during the last 5000 years in China. Scientia Sinica **16**, 226–256 (1973).

[11] U. Büntgen , 2500 years of European climate variability and human susceptibility. Science **331**, 578–582 (2011). 21233349 10.1126/science.1197175

[12] F. H. Chen , East Asian summer monsoon precipitation variability since the last deglaciation. Sci. Rep. **5**, 11186 (2015). 26084560 10.1038/srep11186 PMC4471663

[13] J. Yin, Y. Su, X. Fang, Relationships between temperature change and grain harvest fluctuations in China from 210 BC to 1910 AD. Quat. Int. **355**, 153–163 (2015).

[14] F. H. Chen , Holocene moisture and East Asian summer monsoon evolution in the northeastern Tibetan Plateau recorded by Lake Qinghai and its environs: A review of conflicting proxies. Quaternary Sci. Rev. **154**, 111–129 (2016).

[15] E. Liang , The 1920S drought recorded by tree rings and historical documents in the semi-arid and arid areas of Northern China. Clim. Change **79**, 403–432 (2006).

[16] B. Yang, M. He, L. Yang, F. Wang, F. C. Ljungqvist, Pine maximum latewood density in semi-arid northern china records hydroclimate rather than temperature. Geophysic. Res. Lett. **50**, e2023GL104362 (2023).

[17] Z. Hao, M. Bai, D. Xiong, Y. Liu, J. Zheng, The severe drought of 1876–1878 in North China and possible causes. Clim. Change **167**, 1–17 (2021).34248235

[18] B. Yang , A 3,500-year tree-ring record of annual precipitation on the northeastern Tibetan Plateau. Proc. Natl. Acad. Sci. U.S.A. **111**, 2903–2908 (2014). 24516152 10.1073/pnas.1319238111 PMC3939907

[19] X. M. Shao , Climatic implications of a 3585-year tree-ring width chronology from the northeastern Qinghai-Tibetan Plateau. Quaternary Sci. Rev. **29**, 2111–2122 (2010).

[20] Q. B. Zhang, G. Cheng, T. Yao, X. Kang, J. Huang, A 2,326-year tree-ring record of climate variability on the northeastern Qinghai-Tibetan Plateau. Geophys. Res. Lett. **30**, 1739 (2003).

[21] B. Yang , Long-term decrease in Asian monsoon rainfall and abrupt climate change events over the past 6,700 years. Proc. Natl. Acad. Sci. U.S.A. **118**, e2102007118 (2021). 34282014 10.1073/pnas.2102007118 PMC8325342

[22] Y. F. Shi , Recent and future climate change in northwest china. Clim. Change **80**, 379–393 (2007).

[23] B. Yang, Evidence for a late Holocene warm and humid climate period and environmental characteristics in the arid zones of northwest China during 2.2 ∼1.8 kyr B.P. J. Geophys. Res. **109**, D02105 (2004).

[24] Z. Wang , A high-resolution stalagmite record from Luoshui Cave, Central China over the past 23.5 kyr. Quaternary Sci. Rev. **282**, 107443 (2022).

[25] A. G. Bunn, E. Jansma, M. Korpela, R. D. Westfall, J. Baldwin, Using simulations and data to evaluate mean sensitivity (ζ) as a useful statistic in dendrochronology. Dendrochronologia **31**, 250–254 (2013).

[26] C. Xu , Tree-ring oxygen isotope across monsoon Asia: Common signal and local influence. Quatern. Sci. Rev. **269**, 107156(2021).

[27] E. R. Cook, P. J. Krusic, Program ARSTAN: A tree-ring standardization program based on detrending and autoregressive time series modeling, with interactive graphics (Columbia University, Palisades, NY, 2005).

[28] Y. Liu , Anthropogenic aerosols cause recent pronounced weakening of Asian summer monsoon relative to last four centuries. Geophys. Res. Lett. **46**, 5469–5479 (2019).

[29] D. E. Zhang, A Compendium of Chinese Meteorological Records of the Last 3,000 Years (Jiangsu Education Press, Nanjing, 2013).

[30] H. von Storch , Reconstructing past climate from noisy data. Science **306**, 679–682 (2004). 15459344 10.1126/science.1096109

[31] E. A. Vaganov, M. K. Hughes, A. V. Shashkin, Growth Dynamics of Conifer Tree Rings: Images of Past and Future Environments (Springer Science & Business Media, 2006).

[32] F. Chen , Asian dust-storm activity dominated by Chinese dynasty changes since 2000 BP. Nat. Commun. **11**, 992 (2020). 32080182 10.1038/s41467-020-14765-4 PMC7033097

[33] J. Liu , Spatiotemporal characteristics, patterns, and causes of land-use changes in China since the late 1980s. J. Geogr. Sci **24**, 195–210 (2014).

[34] G. C. Xu, H. Yao, Historical climate changes of the West China in the Holocene. Adv. Water Resour. **2**, 277–288 (1991).

[35] H. Iizuka , Reconstruction of drought and long-rain chronologies since the 17th century in Central Japan using intra-annual tree-ring oxygen isotope ratios and documentary records. EGUsphere **2024**, 1–19 (2024).

[36] D. E. Zhang, Historical Records of environmental changes and agricultural development in Northwest China. Adv. Clim. Change Res. **1**, 58–64 (2005).

[37] Z. J. Wang, A historic investigation of climate change in the Qin and Han Dynasties. Histor. Res. **2**, 3–19 (1995).

[38] P. Y. Zhang, Historical Climate Change in China (Shandong Science & Technology Press, Ji’nan, 1996).

[39] P. K. Wang , Construction of the REACHES climate database based on historical documents of China. Sci. Data **5**, 180288 (2018). 30561430 10.1038/sdata.2018.288 PMC6298253

[40] L. Yang, Z. Shi, S. Zhang, H. F. Lee, Climate change, geopolitics, and human settlements in the hexi corridor over the Last 5,000 Years. Acta Geologica Sinica **94**, 612–623 (2020).

[41] D. Zhang, H. Deng, Historical human activities accelerated climate-driven desertification in China’s Mu Us Desert. Sci. Total Environ. **708**, 134771 (2020). 31784165 10.1016/j.scitotenv.2019.134771

[42] J. Zheng , How climate change impacted the collapse of the Ming dynasty. Clim. Change **127**, 169–182 (2014).

[43] X. Fang, Y. Su, J. Yin, J. Teng, Transmission of climate change impacts from temperature change to grain harvests, famines and peasant uprisings in the historical China. Sci. China, Ser. D Earth Sci. **58**, 1427–1439 (2015).

[44] J. Yin, Y. H. Luo, X. Q. Fang, Y. SU, The climatic and harvest backgrounds of dynastic flourishing ages and transitions in China during 210 BC to 960 AD. J. Earth Environ. **5**, 400–409 (2014).

[45] Q. Feng , Domino effect of climate change over two millennia in ancient China’s Hexi Corridor. Nat. Sustainability **2**, 957–961 (2019).

[46] Q. S. Ge, H. L. Liu, J. Y. Zheng, L. B. Xiao, The climate change and social development over the last two millennia in China. Chinese J. Nat. **35**, 9–021 (2013).

[47] X. Q. Fang , The Social Impacts of Climate Change in China Over the Past 2000 Years (Springer, 2024).

[48] F. Chen , Discussion of the “warming and wetting” trend and its future variation in the drylands of Northwest China under global warming. Sci. China Earth Sci. **66**, 1241–1257 (2023).

[49] B. Wang, R. Wu, Interannual variability of the Asian summer monsoon: Contrasts between the Indian and the Western North Pacific-East Asian Monsoons. J. Clim. **14**, 4073–4090 (2001).

[50] B. Yang , Drought variability at the northern fringe of the Asian summer monsoon region over the past millennia. Clim. Dynam. **43**, 845–859 (2014).

[51] Z. Wu, B. Wang, J. Li, F. Jin, An empirical seasonal prediction model of the east Asian summer monsoon using ENSO and NAO. J. Geophys. Res. **114**, D18120 (2009).

[52] S. H. Baek , Precipitation, temperature, and teleconnection signals across the combined North American, Monsoon Asia, and old world drought atlases. J. Clim. **30**, 7141–7155 (2017). 30449951 10.1175/JCLI-D-16-0766.1 PMC6235455

[53] R. J. Francey, G. D. Farquhar, An explanation of ^13^C/^12^C variations in tree rings. Nature **297**, 28–31 (1982).

[54] D. McCarroll, N. J. Loader, Stable isotopes in tree rings. Quatern. Sci. Rev. **23**, 771–801 (2004).

[55] A. Gessler , Stable isotopes in tree rings: Towards a mechanistic understanding of isotope fractionation and mixing processes from the leaves to the wood. Tree Physiol. **34**, 796–818 (2014). 24907466 10.1093/treephys/tpu040

[56] J. S. Roden, G. G. Lin, J. R. Ehleringer, A mechanistic model for interpretation of hydrogen and oxygen isotope ratios in tree-ring cellulose. Geochim. Cosmochim. Acta **64**, 21–35 (2000).

[57] A. Gessler, K. Treydte, The fate and age of carbon–insights into the storage and remobilization dynamics in trees. New Phytol. **209**, 1338–1340 (2016). 26840248 10.1111/nph.13863

[58] K. Treydte , Seasonal transfer of oxygen isotopes from precipitation and soil to the tree ring: Source water versus needle water enrichment. New Phytol. **202**, 772–783 (2014). 24602089 10.1111/nph.12741

[59] I. Labuhn , Tree age, site and climate controls on tree ring cellulose δ^18^O: A case study on oak trees from south-western France. Dendrochronologia **32**, 78–89 (2014).

[60] T. Nakatsuka , A 2600-year summer climate reconstruction in central Japan by integrating tree-ring stable oxygen and hydrogen isotopes. Clim. Past **16**, 2153–2172 (2020).

[61] T. Wieloch, G. Helle, I. Heinrich, M. Voigt, P. Schyma, A novel device for batch-wise isolation of alpha-cellulose from small-amount wholewood samples. Dendrochronologia **29**, 115–117 (2011).

[62] S. Kang , Tree-ring stable carbon isotope as a proxy for hydroclimate variations in semi-arid regions of North-Central China. Forests **13**, 492 (2022).

[63] N. J. Loader, I. Robertson, A. C. Barker, V. R. Switsur, J. S. Waterhouse, An improved technique for the batch processing of small wholewood samples to α-cellulose. Chem. Geol. **136**, 313–317 (1997).

[64] G. H. F. Young, N. J. Loader, D. McCarroll, A large scale comparative study of stable carbon isotope ratios determined using on-line combustion and low-temperature pyrolysis techniques. Palaeogeogr. Palaeoclimatol. Palaeoecol. **300**, 23–28 (2011).

[65] G. H. F. Young , Oxygen stable isotope ratios from British oak tree-rings provide a strong and consistent record of past changes in summer rainfall. Clim. Dyn. **45**, 3609–3622 (2015).

[66] D. McCarroll , Correction of tree ring stable carbon isotope chronologies for changes in the carbon dioxide content of the atmosphere. Geochim. Cosmochim. Acta **73**, 1539–1547 (2009).

[67] T. M. Wigley, K. R. Briffa, P. D. Jones, On the average value of correlated time series, with applications in dendroclimatology and hydrometeorology. J. Clima. Appl. Meteor. **23**, 201–213 (1984).

[68] F. Biondi, K. Waikul, DENDROCLIM2002: A C++ program for statistical calibration of climate signals in tree-ring chronologies Comput. Geosci. **30**, 303–311 (2004).

[69] H. Du , A comparison of pre-Millennium eruption (946 AD) and modern temperatures from tree rings in the Changbai Mountain, northeast Asia. CliPD **2022**, 1–32 (2022).

[70] S. G. Dee , No consistent ENSO response to volcanic forcing over the last millennium. Science **367**, 1477–1481 (2020). 32217726 10.1126/science.aax2000

[71] A. J. Kirchhefer, “A discontinuous tree-ring record AD 320–1994 from Dividalen, Norway: Inferences on climate and treeline history” in Mountain Ecosystems: Studies in Treeline Ecology, (Springer, 2005), pp. 219–235.

[72] K. M. Cobb, C. D. Charles, H. Cheng, R. L. Edwards, El Niño/Southern Oscillation and tropical Pacific climate during the last millennium. Nature **424**, 271–276 (2003). 12867972 10.1038/nature01779

[73] J. P. Cropper, Tree-ring skeleton plotting by computer. Tree Ring Bullet. **39**, 47–60 (1979).

[74] V. V. Shishov , VS-oscilloscope: A new tool to parameterize tree radial growth based on climate conditions. Dendrochronologia **39**, 42–50 (2016).

[75] H. Fritts, Tree Rings and Climate (Elsevier, ACM Transactions on Applied Perception, 1976).

[76] C. Qin , Data from “Persistent humid climate favored the Qin and Western Han Dynasties in China around 2200 y ago.” NOAA. https://www.ncei.noaa.gov/access/paleo-search/study/39724. Deposited 2 August 2024. 10.1073/pnas.2415294121 PMC11725930 39715434

[77] C. Qin , Data from “Persistent humid climate favored the Qin and Western Han Dynasties in China around 2200 y ago.” NOAA. https://www.ncei.noaa.gov/access/paleo-search/study/39725. Deposited 2 August 2024. 10.1073/pnas.2415294121 PMC11725930 39715434

[78] C. Qin , Data from “Persistent humid climate favored the Qin and Western Han Dynasties in China around 2200 y ago.” NOAA. https://www.ncei.noaa.gov/access/paleo-search/study/39726. Deposited 2 August 2024. 10.1073/pnas.2415294121 PMC11725930 39715434

