# Supplementary material for: Persistent humid climate favored the Qin and Western Han Dynasties in China around 2,200 y ago
Source: Proc Natl Acad Sci U S A. 2024 Dec 23;122(1):e2415294121. doi: 10.1073/pnas.2415294121 (PMC11725930; doi:10.1073/pnas.2415294121)
Supplement: Supplementary file 1 — Appendix 01 (PDF) [file pnas.2415294121.sapp.pdf]

PNAS

Supporting Information for

Persistent humid climate favored the Qin and Western Han Dynasties in China around 2200 years ago

Chun Qin, Bao Yang, Achim Bräuning, Fredrik Charpentier Ljungqvist, Timothy J. Osborn, Vladimir Shishov, Minhui He, Shuyuan Kang, Lea Schneider, Jan Esper, Ulf Büntgen, Jussi Grießinger, Danqing Huang, Peng Zhang, Stefanie Talento, Elena Xoplaki, Jürg Luterbacher & Nils Chr. Stenseth

Corresponding author: Nils Chr. Stenseth or Bao Yang
Email: n.c.stenseth@mn.uio.no or yangbao@nju.edu.cn

This PDF file includes:

Supplementary text
Figures S1 to S24
Tables S1 to S11
SI References

Supporting Information Text

Identifying archeological wood tree species. According to wood anatomical characteristics, the archeological wood (AW) selected for this study was first classified as conifer, given that the wood tissues were only composed of tracheids, without vessels (pores). Resin canals were observed in transverse and tangential sections, confirming that samples were *Pinus* (not *Juniperus*) for biogeographical reasons, since no other conifer species are distributed in this area. A closer examination of the transverse, radial, and tangential sections of the wood samples provided further evidence for determining the wood species. In the transverse section (Fig. S2a), the shape of earlywood tracheids was mainly rectangular, squared, or polygonal, while that of latewood tracheids was mainly rectangular or circular. The transition from earlywood to latewood was gradual. Large resin canals with an average of 8 to 15 thin-walled epithelial cells were observed in the transverse section. With respect to the tangential section (Fig. S2b), two types of xylem rays were identified, either uniseriate rays comprised of 2 to 15 cells in height, or fusiform rays comprised of horizontal resin canals. The two ends of the rhombic-shaped resin canals were both uniseriate, and the average ray height was 2 to 10 cells. Axial tracheids with bordered pits were almost uniseriate in the radial section (Fig. S2c). The cross-field pits were fenestriform. The geographic location of the wood samples (Jingyuan County, northwestern China) combined with their anatomical features thus constrains the potential species as either *Pinus bungeana* Zucc. ex Endl. or *Pinus tabulaeformis* Carr. (1). We compared the anatomical features of AW samples with those of living *P. tabulaeformis* Carr. (LP) specimens (Fig. S2d, e, f) in Jingyuan County, observing close similarities between their transverse, tangential, and radial sections. Consequently, we identified the sample species as Chinese pine (*P. tabulaeformis* Carr.).

Provenance of historical timbers. Huangwan tomb likely served as the burial site for residents of Zhanyin city (located at Yagoushui Village, Shuiquan Town, Baiyin City, Gansu Province) (2). As shown in Fig.S21, both Zhanyin city (1470 m a.s.l.) and Huangwan tomb (1394 m a.s.l.) are proximate to the Yellow River.

Historical documents indicate abundant local forests (2, 3) and no records of distant timber importation, suggesting a local source for construction uses. There was also extensive military-led logging of forest in the mountains (4-6). As mentioned above, the tree species used in the Huangwan tomb is Pinus tabulaeformis, known for its drought and cold resistance, primarily found at 2150-2450 meters in Jingyuan County. Hasi Mountain represents the only site where natural forests of *Pinus tabulaeformis* grow within 100 kilometers of the Huangwan tomb. The vegetation of Hasi Mountain is composed of low-altitude *Pinus tabulaeformis* forest, high-altitude Picea crassifolia forest and a small amount of *Betula platyphylla* and *Populus davidiana* (6). There are three possible overland routes to transport timbers for Huangwan tomb construction, as indicated by the documents (4, 6) and topographic maps.

Dating living Chinese pine. A ring-width chronology based on living Chinese pine (LP) samples was established through international standard procedures for tree-ring dating on the TSAP-Win platform (scientific version) (7). The software COFECHA was used for the quality control of these series (8).

Dating of the archeological wood. It was not possible to establish an absolutely dated chronology using AW samples alone due to the lack of reliable long-term reference chronologies in this region. Instead, we used three independent lines of evidence to estimate the dates of these samples.

1) Historical dating. Background dating was provided using concomitant tomb relics. The vertical-hole, wooden-coffin tomb structure suggested that it was most probably constructed prior to the mid-Han Dynasty. The “Han Banliang” coins and the “Han Li” calligraphy in the manuscripts found in the tombs were further evidence of the Han Dynasty (from 202 BCE to 220 CE). This has been confirmed by two surveys conducted by the National Cultural Heritage Administration in 1976 and 2010 CE, respectively. The latest findings indicate that these tombs were constructed in the historically renowned “Zhanyin” or “Chunyin” city, which first came into existence between 214 BCE and 111 CE (2). Accordingly, the cut-off dates of the AW samples are between 202 BCE and 111 CE. The wood samples were used for dating the year of the tomb construction (9). Due to decomposition, the outermost rings of these samples were unavailable. Therefore, the remaining chronology was likely contemporaneous with the Common Era (CE) or earlier.

2) Dendrochronological dating. Due to the limited number of AW samples available, each wood block was measured in several radial directions to detect potential false or missing rings. A mean ring-width curve for each wood block, calculated from 2–3 different radial measurements, was established for each tree. Prior to dating the samples, we built a floating chronology based on AW samples via cross-dating on the same TSAP-Win platform as employed for the living Chinese pine. To obtain absolute dating, the nearest long-term chronology (the QLS chronology, located nearly 600 km from the sampling site) (10) was chosen as the reference dating chronology in this study. The QLS chronology represents precipitation variability across a large part of northern China during the past 3500 years. The dating strategy was to retain the dating results according to the above reliable historical dating period. Unfortunately, it was impossible to achieve unambiguous dendrochronological dating due to poor pattern matching between the floating chronology and the QLS reference chronology (likely due to the 600 km distance between the two sites). Therefore, single mean curve dating was applied as an additional method, which focused first on the subset of trees with the strongest cross-dating signal between single AW series and the QLS reference chronology. From a total of 12 trees that fell into the previous historical dating period, eight trees shared a common pattern in the floating chronology via the five best-match positions as the program’s default setting. We built a provisional chronology comprising these eight trees, and cross-dated the other 18 samples to build the final chronology. Only the dating result from 336 BCE to 49 BCE fell into the previous historical dating period (Fig. S3). Both visual growth pattern matching and statistical tests in the software package TSAP (7) indicated that the dating results are reliable (Fig. S3). Next, we fitted the other samples into the provisional chronology. The final AW chronology, an anchored form of the provisional chronologies, covered the period from 336 BCE to 31 BCE.

3) Radiocarbon dating verification. Radiocarbon dating was employed to verify the above dating approach. We selected one wood sample (JGP040), which showed no distortions and no missing rings, and cut it year

by year from pith to bark. One out of every 10 tree rings was then selected for the ^{14}C dating, i.e. the 1st, 11st, 21st, ……, 141st rings, respectively. Prior to the ^{14}C analysis, the 15 samples were subjected to an acid-alkali-acid treatment that removed contaminants (carbonates and humic acids) (11). Finally, the samples were ^{14}C dated based on INTCAL13 (12), following the Standard AMS Delivery Service provided by Beta Analytic Inc. (Miami, Florida, United States). The mathematics used for the calibration procedure followed Talma et al. (13). All 15 samples were also dendrochronologically dated. As shown in Fig. S4, 12 out of 15 dendrochronological sample dates fell within the range of the respective radiocarbon age; this is not surprising considering that radiocarbon dating is subject to a plateau effect during this period. Overall, the dendrochronological dates were supported by the ^{14}C dates, within an error band of ±30 years.

Chronology comparison and climate sensitivity. The reliable periods of the final chronologies covering the Qin–Western Han dynasties and the present day extended from 282–50 BCE and 1695–2015 CE for TRW, and 270–77 BCE and 1751–2015 ($\delta^{13}C$) / 2010 ($\delta^{18}O$) CE for isotopes (Fig. 3). The subsequent analysis focuses on the common credible periods of three tree-ring parameters, specifically from 270–77 BCE and 1751–2015 CE.

Pearson's correlation coefficients between the standard ring-width chronology (TRW) and $\delta^{13}C$ record for the period 1751–2015 CE (270–77 BCE) are -0.42 (-0.48), $p<0.01$; between TRW and $\delta^{18}O$ they are -0.004 (-0.26), $p>0.05$ ($p<0.01$); and between $\delta^{13}C$ and $\delta^{18}O$ they are 0.30 (0.50), $p<0.01$. Good agreement between $\delta^{13}C$ and $\delta^{18}O$, and between $\delta^{13}C$ and TRW suggests that these proxies have common dominant climate controls and that their combination can offer more information on climatic variability than that of individual series.

Previous studies suggest that tree-ring cellulose $\delta^{13}C$ is related to relative humidity (rH) in the study region (14, 15), while tree-ring $\delta^{18}O$ is associated with summer rH and $\delta^{18}O$ in precipitation and soil moisture (16-19). The TRW chronology is significantly correlated with the summer (current June to August: C6-8) self-calibrating Palmer Drought Severity Index (scPDSI) (r=0.59) and precipitation (r=0.49). The $\delta^{13}C$ data respond most strongly to summer (June-August) scPDSI (r=-0.58), rH (r=-0.59) and temperature (May-August, r=0.60), and to annual (P8-C7) precipitation (r=-0.57). The strong link between $\delta^{13}C$ and temperature partly originates from coherent long-term trends, and the correlation decreases to r=0.44 when first-order differenced. The $\delta^{18}O$ data contain a weaker extended summer (C7-C10) scPDSI signal (r=-0.36). Among the three tree-ring parameters, hydroclimatic signals are most strongly captured by $\delta^{13}C$ and least strongly by $\delta^{18}O$, confirming that $\delta^{13}C$ is mostly controlled by rH via stomatal conductance, while $\delta^{18}O$ is additionally affected by the variability of the source water $\delta^{18}O$ signal.

The VS model predicts that radial growth increases by 10% with a precipitation rise of 10% (23 mm), whereas growth decreases by nearly 2% if temperature rises by 1°C (Fig. S18). This implies that that the radial growth of trees in this region is more sensitive to precipitation than to temperature, consistent with the results from He et al. (20) and supporting our interpretation that the difference in measured mean ring widths between AW and LP samples arose from a change in precipitation rather than temperature.

Ring-width altitude sensitivity analysis. A large number of historical documents show that during the Qin–Western Han period, humans in the region began to cut down trees on a large scale for houses, tombs, firewood, etc. From the wood collected in this study, there are 6 trees with an age of less than 100 years, and 19 trees with an age of more than 150 years. It seems that people selectively tend to collect larger trees possibly either from lower or higher elevations considering typical narrow altitudinal range of the forest stands with only 300 meters. It is also noted that the onset of large-scale artificial tending (clearing fallen trees, diseased and insect-infested trees, rotten trees, thinning) in 1958 (3) triggered a more substantial growth release at lower elevations. This effect was further amplified by intensive felling activities during the period from 1965 to 1972 (3). The growth release exhibited distinct patterns at different altitudes, with more pronounced effects at lower elevations (Fig. S22).

Taking all the above into account, it is reasonable to calculate mean growth rates as a function of ring biological age by sampling living trees from all the elevations (2137–2456m a.s.l.) to facilitate comparison with those from archaeological tree samples (Fig. S5). In this process, we also added 36 new tree samples from the low elevations. Unfortunately, it was impossible to sample the old *Pinus tabulaeformis* at low elevations due to intensive logging activities during 1968–1972 CE and extensive artificial cultivation starting in 1958 CE, which led to significant growth releases (Fig. S22) at the lower forest boundary.

Considering the possible altitude effects between AW and LP samples, and the uncertainty caused by the recent growth release of low-altitude trees, we regard the growth rates at each growth stage from 1 to 220 years (sample depth⩾5 trees and relatively stable) as a reasonable range. It means the ring-width is wider by 22%~30% in Qin-Western Han Dynasties than at present. Based on this result, the increased precipitation during Qin-Western Han is +18%- 34% higher than at present. This result is also consistent with our $\delta^{13}C$-based precipitation reconstruction (16%±6%).

$\delta^{13}C$ measurement validation and altitude sensitivity analysis. The two sets of $\delta^{13}C$ data derived from two different laboratories of Germany and UK showed significant (r=0.87, n =92, p<0.001) correlation between the $\delta^{13}C$ records (Fig. S23). The measured samples and methods used (individual & pooled approach) for these two datasets are completely independent. Considering the analytical precision of the measurement was 0.1‰, the difference in their means is completely acceptable. There is a strong correlation between the measurements between high and low elevations, and no significant difference in mean $\delta^{13}C$ was detectable (Fig. S24). There is also no significant trend between the elevation and the mean value of $\delta^{13}C$ (Fig. S24). To sum up, altitude effects on tree-ring $\delta^{13}C$ were examined and found to be negligible. Thus, although we do not know at which altitude the archaeological tree samples were taken, we can make past and present comparisons.

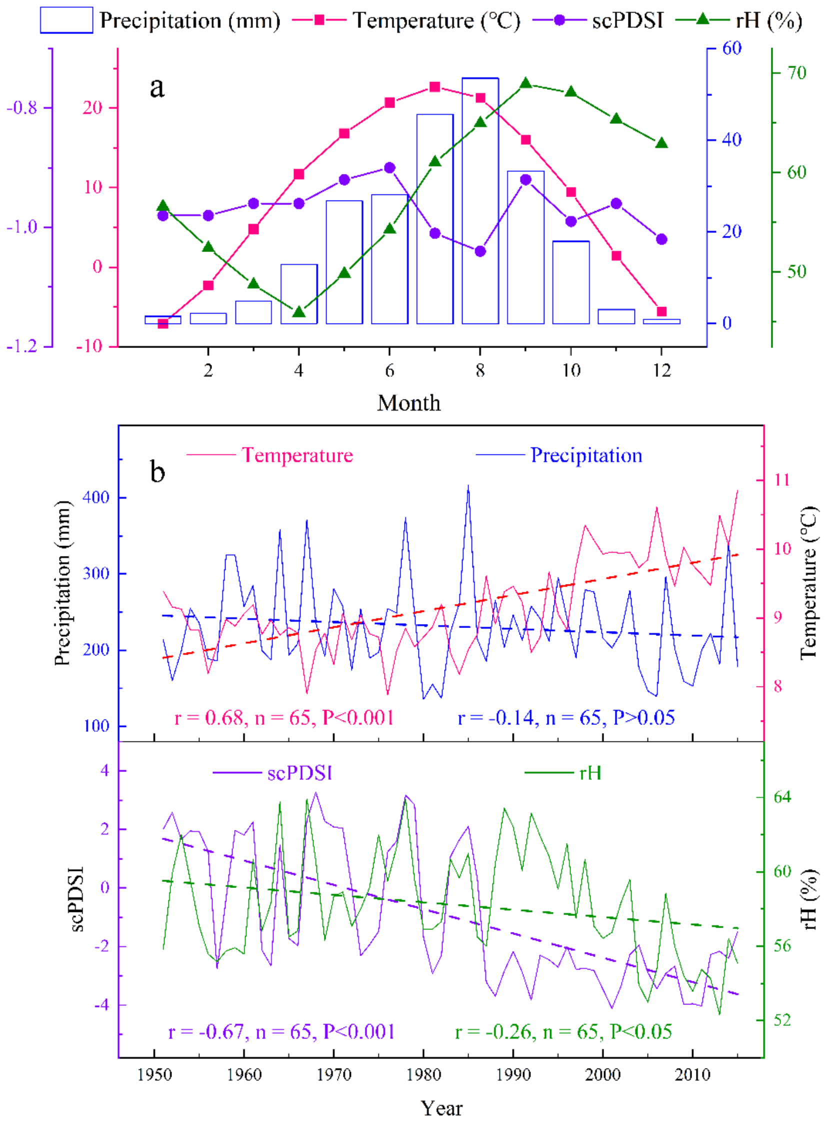

Fig. S1. Recent climatic context for the study area. Average monthly (a) and time-series and their trends of annual (b) mean temperature, precipitation, and relative humidity (rH) recorded at the Jingyuan meteorological station and the average of the nearest Climatic Research Unit (CRU) self-calibrating Palmer Drought Severity Index (scPDSI) grid points (from ref. 31) for the period 1951–2015 CE.

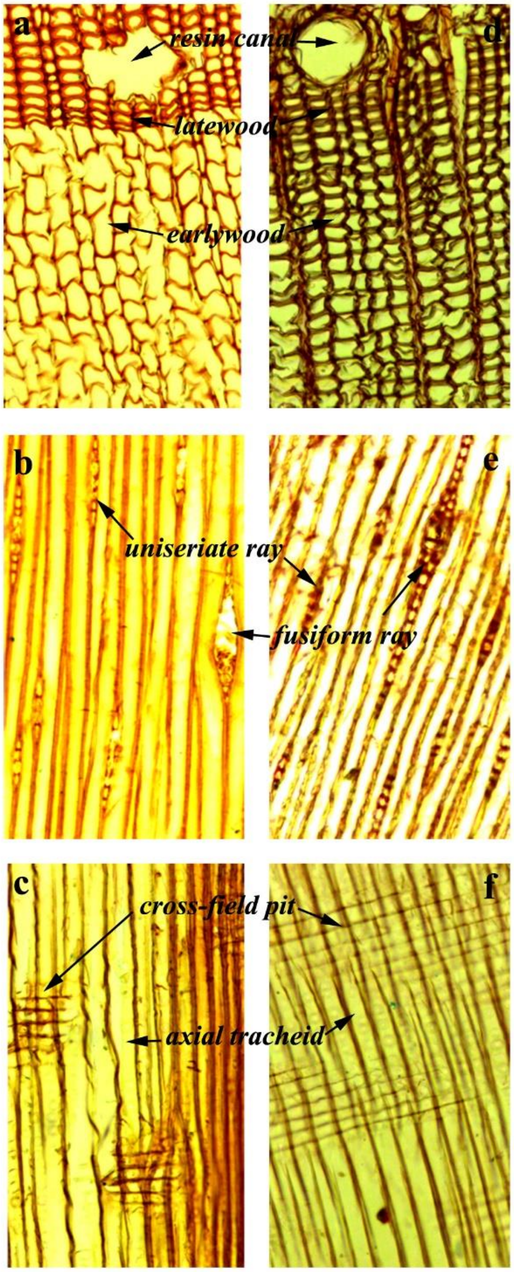

Fig. S2. Anatomical features used to identify the archeological wood tree species. A comparison between anatomical features of archeological wood (AW; left panel: a, b, c) and living Chinese pine (LP; right panel: d, e, f) in Jingyuan county, northwest China. Transverse section (upper panel: a, d), tangential section (middle panel: b, e), and radial section (lower panel: c, f).

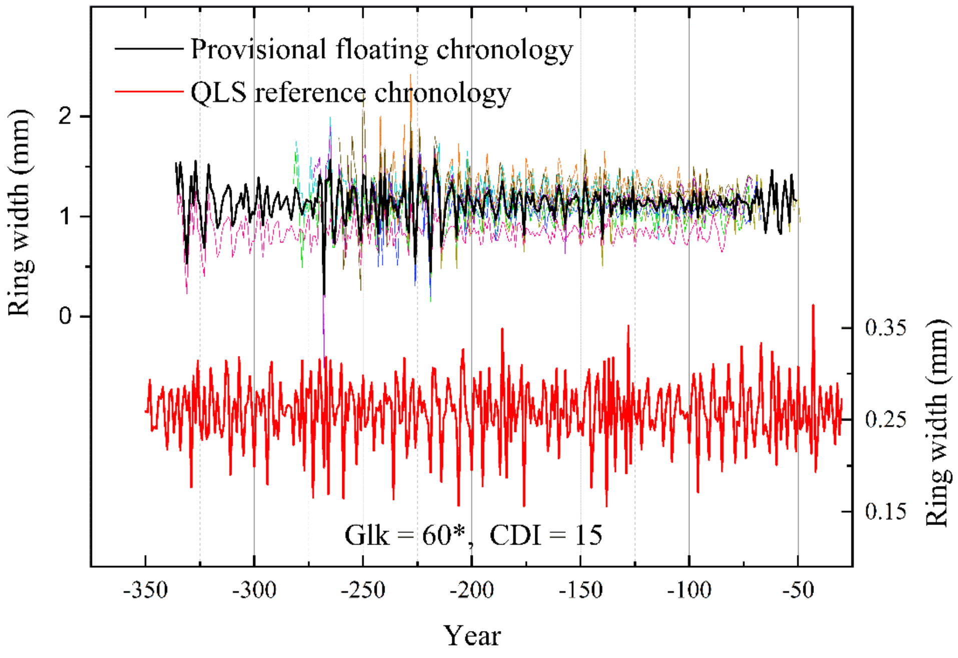

Fig. S3. Cross-dating of an 8-tree subset of the archeological series against the QLS chronology. Comparation between the provisional floating chronology at JY (black bold line) and in the QLS reference chronology (red bold line) after FFT high-pass smoothing with 0.125 year^{-1} cutoff frequency. The thin lines are the tree-ring width (TRW) series of each tree after FFT smoothing. Two indicators of cross-dating agreement are given: Glk (sum of the equal slope intervals in %) with * denoting the value is significant at the α=0.01 level and CDI (cross-dating index) with the value indicating the matching of chronologies.

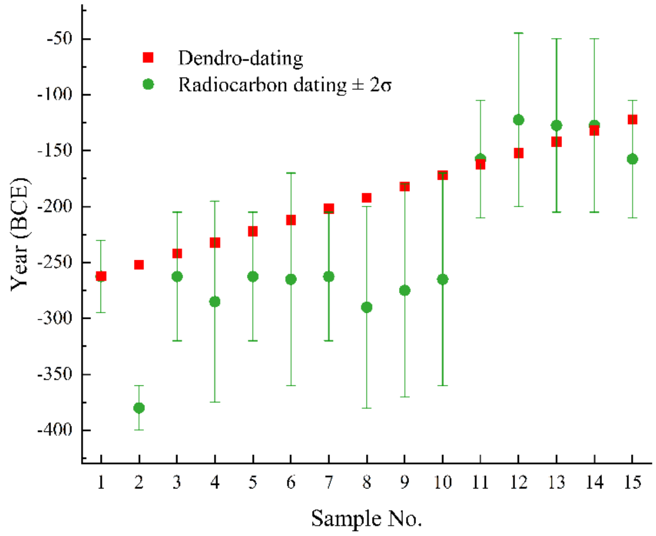

Fig. S4. Comparison of dendrochronological dating and radiocarbon dating results. Sample No. denotes sample codes for the 15 samples taken from tree JGP040.

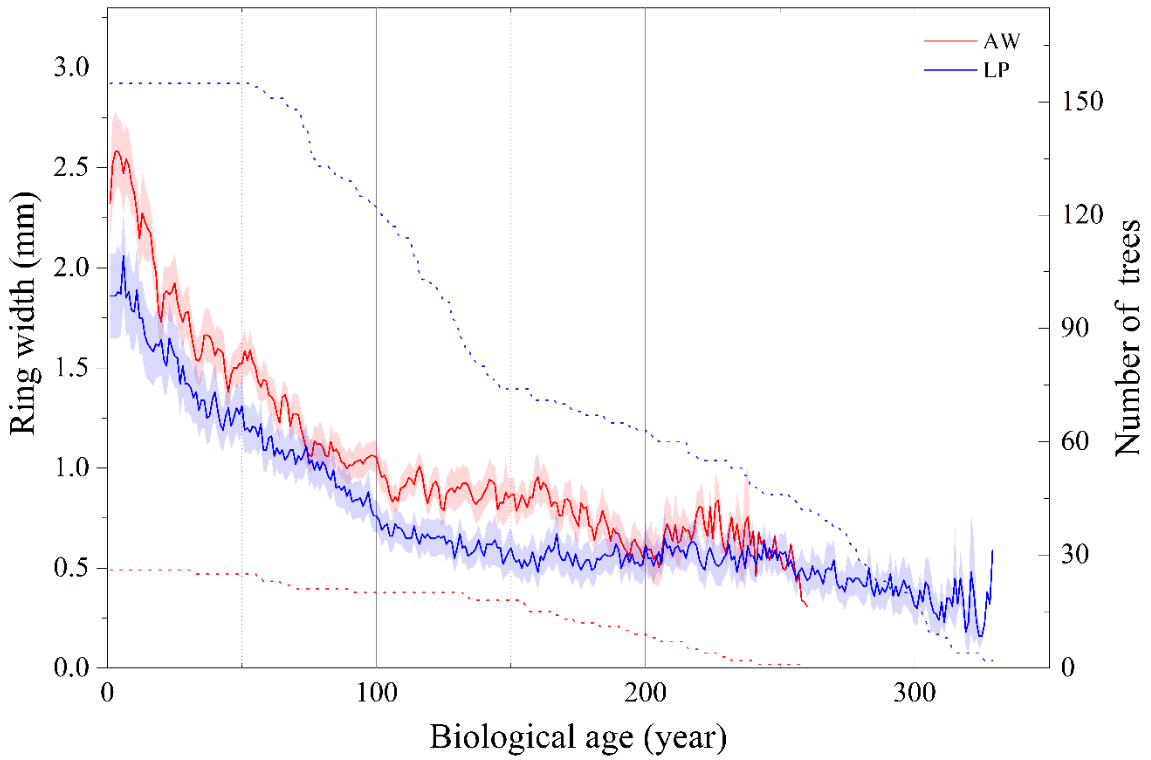

Fig. S5. Comparison of mean growth rates for AW and LP trees as a function of ring biological age. Mean ring-width series (lines) and associated sample depth (numbers of trees, dots) of the archeological wood (AW, red) and living Chinese pine (LP, blue) samples after aligning the data by biological age (years). The blue and red shading denote the 95% confidence ranges for LP and AW, respectively. Sensitivity analysis of calculation methods: We establish tree age-width curves in two ways: 1) first establishing mean curves at three different altitude and then averaging them, 2) directly average them, and found no significant difference in the results. In this study, we calculate the tree age-width sequence by directly averaging them all after sorting them by age.

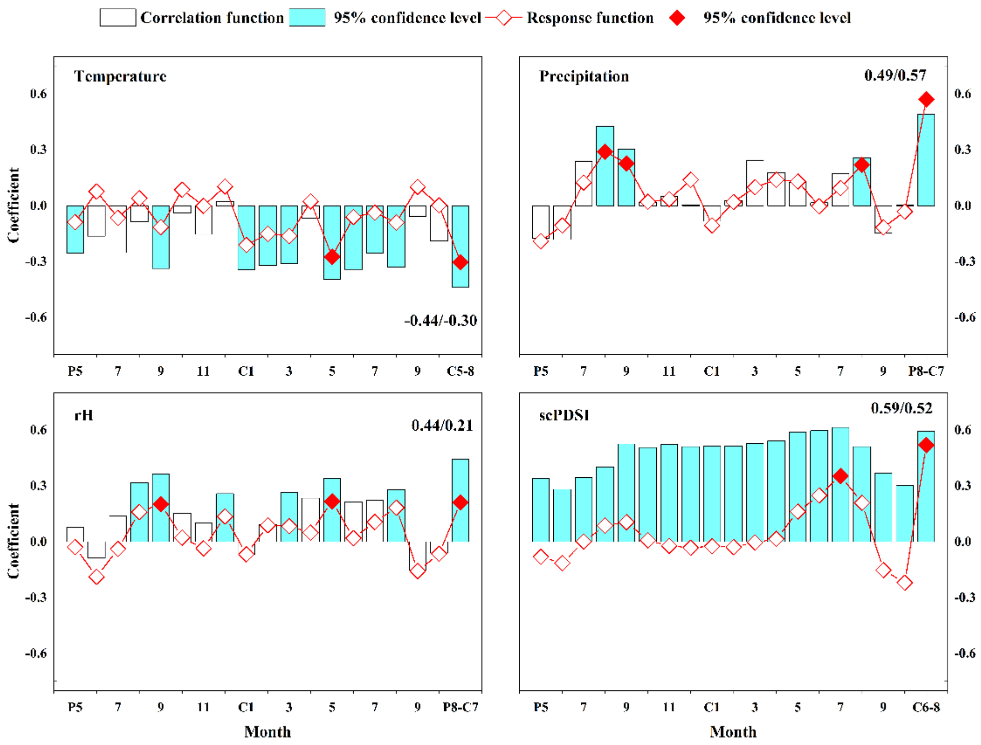

Fig. S6. Identifying the climate signals recorded in the ring-width chronology. Correlations (bars) and response functions (red lines and symbols) between the STD ring-width chronology and monthly mean temperature, precipitation, rH, scPDSI, and the optimal seasonal-average combination during 1951–2015 CE (e.g. P8-C7 denotes the period from the previous August to the current July). Labels, e.g. 0.49/0.57, are the Pearson correlation coefficients for the optimal season using the original values or their first-order differences. Significant values at the 95% confidence level are marked by colored bars and symbols.

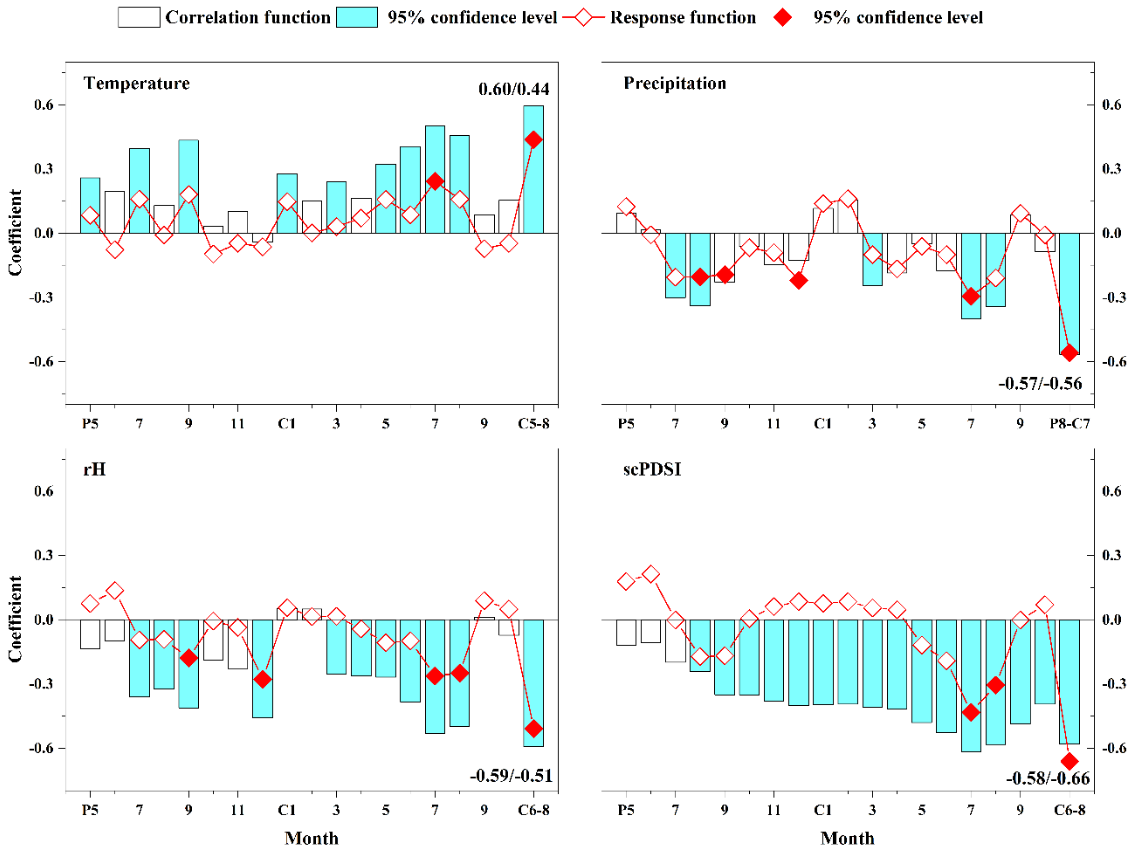

Fig. S7. Identifying climate signals recorded in the δ^{13}C chronology. Correlations (bars) and response functions (red lines and symbols) between the δ^{13}C chronology and the monthly mean temperature, precipitation, rH, scPDSI, and optimal seasonal-average combination during 1951–2015 CE (e.g., C6-8 denotes the period from the current June to August). Labels, e.g., -0.57/-0.56, are the Pearson correlation coefficients of the original sequence and its first-order difference sequence. Significant values at the 95% confidence level are marked by colored bars and symbols.

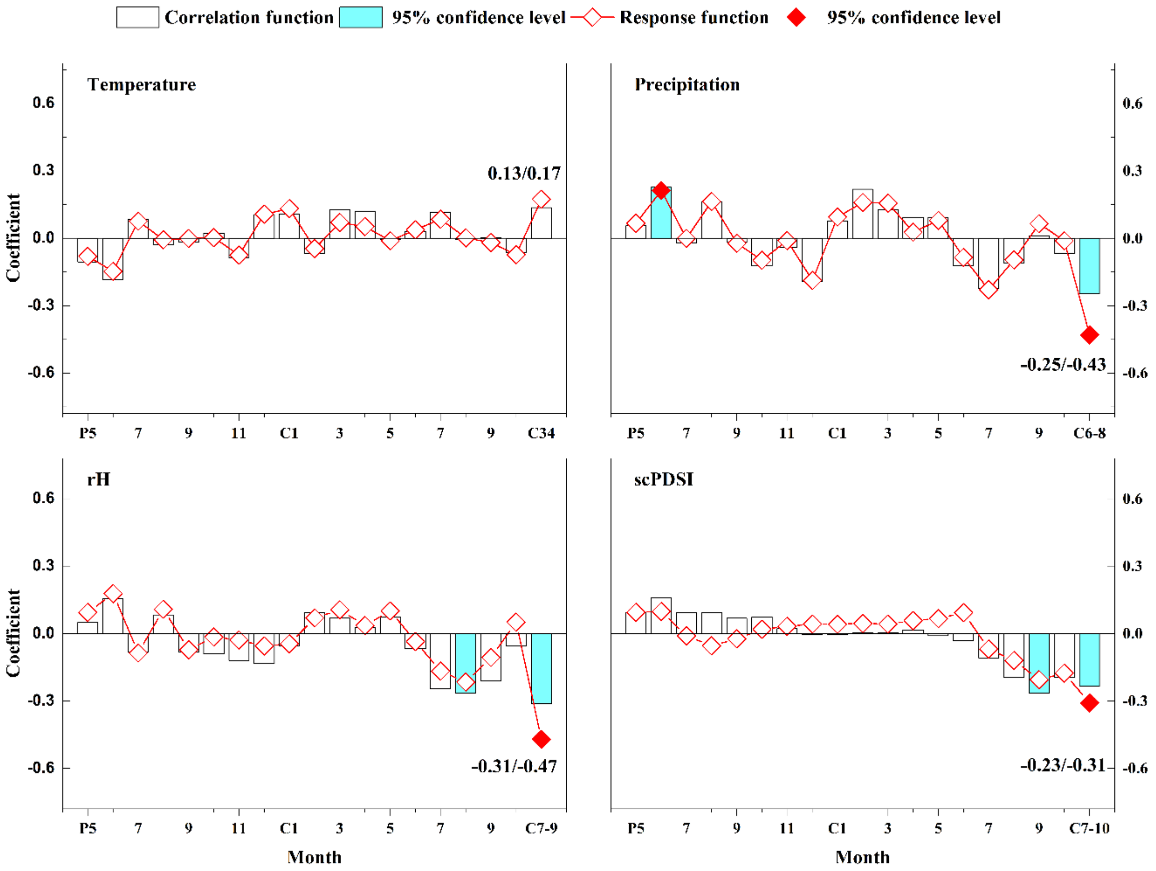

Fig. S8. Identifying climate signals recorded in the δ18O chronology. Correlations (bars) and response functions (red lines and symbols) between $\delta^{18}O$ and the monthly mean temperature, precipitation, rH, scPDSI, and optimal seasonal-average combination during 1951–2010 CE (e.g. C7-9 denotes the period from the current July to September). Labels, e.g. -0.31/-0.47, are the Pearson correlation coefficients of the original sequence and its first-order difference sequence. Significant values at the 95% confidence level are marked by colored bars and symbols.

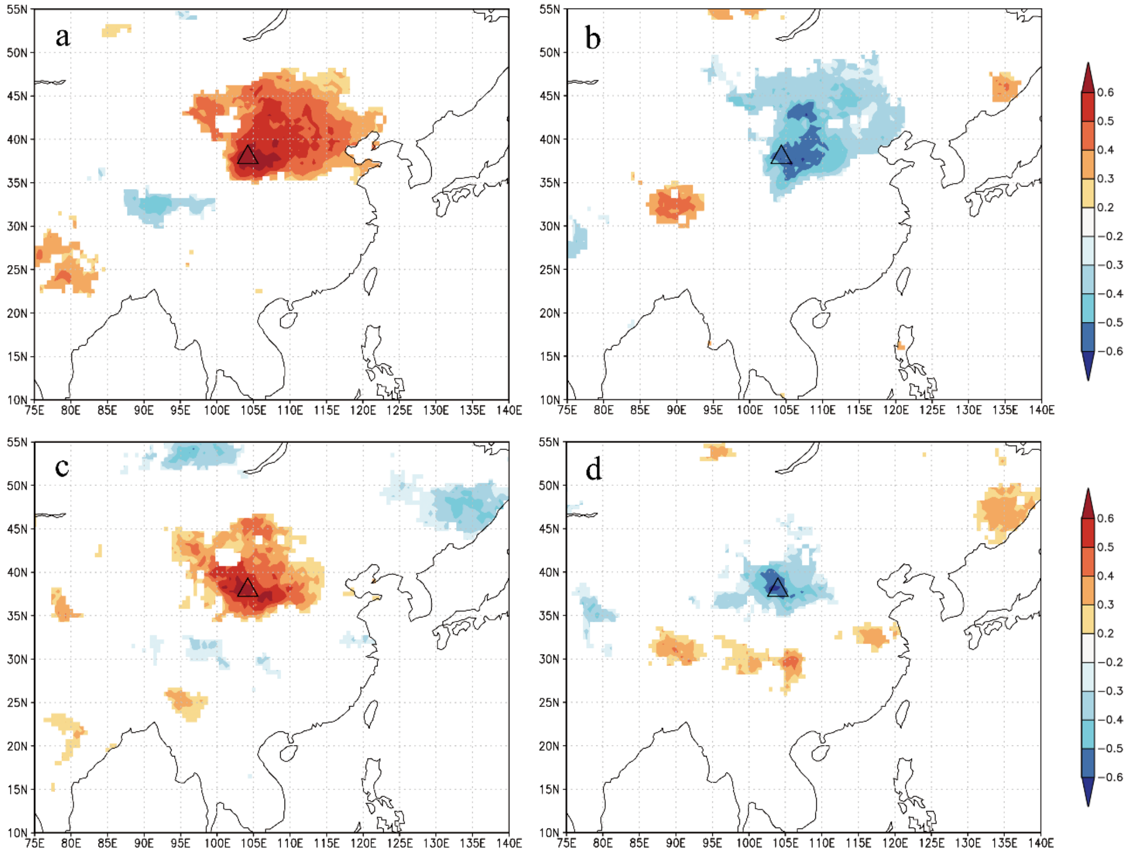

Fig. S9. Spatial representation of drought signals in the tree-ring chronologies. Spatial correlations of ring widths (a), $\delta^{13}C$ (b), and their first order differences (c, d) after removing first-order variations or trends, respectively, using regional gridded scPDSI in P8-C7 during 1951–2015 CE (derived from Climate Explorer, http://climexp.knmi.nl/). The black triangle donates the sampling site.

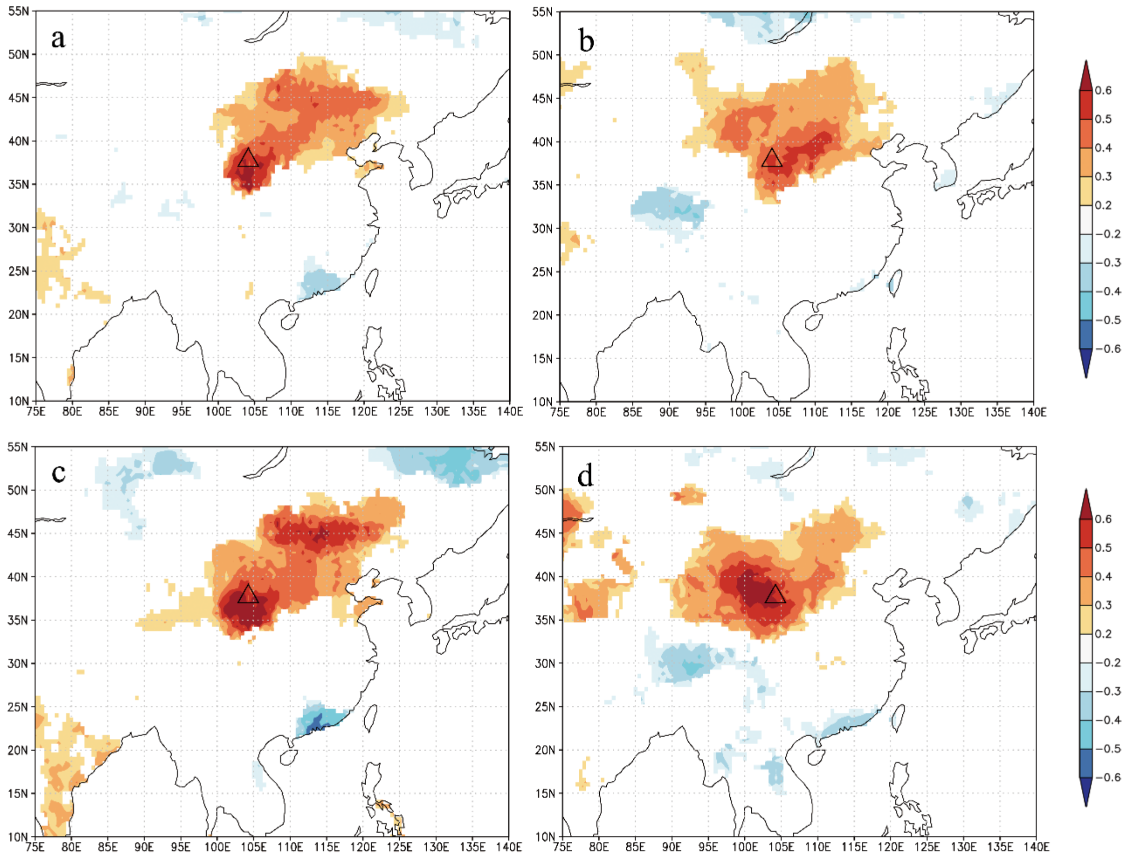

Fig. S10. Spatial representation of precipitation signals from the Jingyuan meteorological station and the $\delta^{13}C$ chronology. Spatial correlations of precipitation in P8-C7 at Jingyuan meteorological station (a), single-regression precipitation reconstruction based on $\delta^{13}C$ in this study (b), and their first order differences (c, d), based on contemporaneous CRU TS 4.08(from ref. 32) precipitation during 1951–2015 CE (derived from Climate Explorer, http://climexp.knmi.nl/). The black triangle indicates the location of the sampling site.

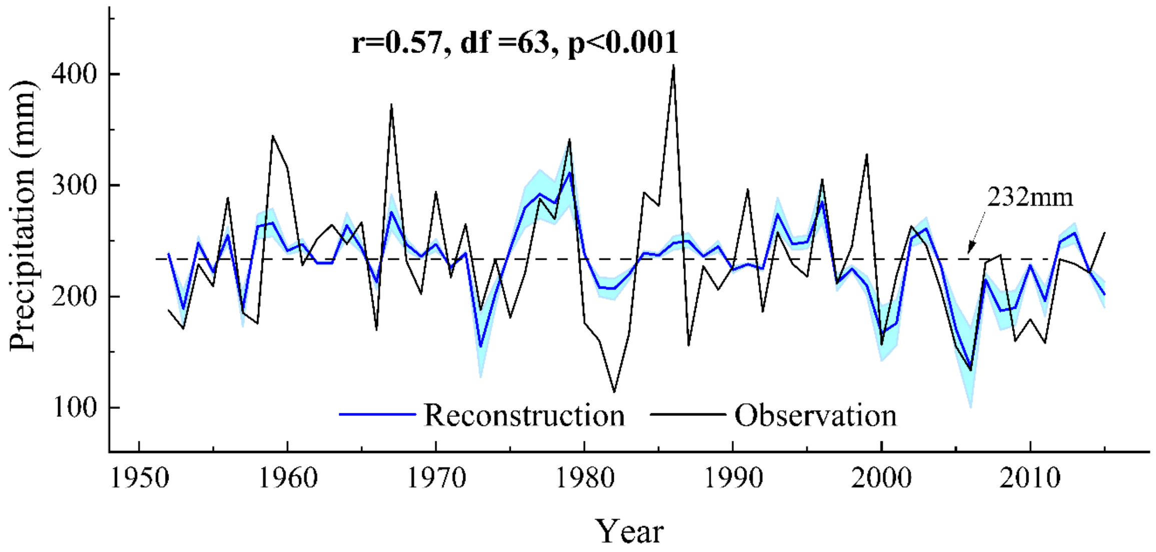

Fig. S11. Comparison between δ^{13}C-based reconstruction (blue line) and the instrumental P8-C7 precipitation record (black line) at Jingyuan station during 1951-2015 CE. The shaded area indicates the 95% confidence level of predicted precipitation. The dashed line and its label are the average of instrumental precipitation records in 1951–2015 CE.

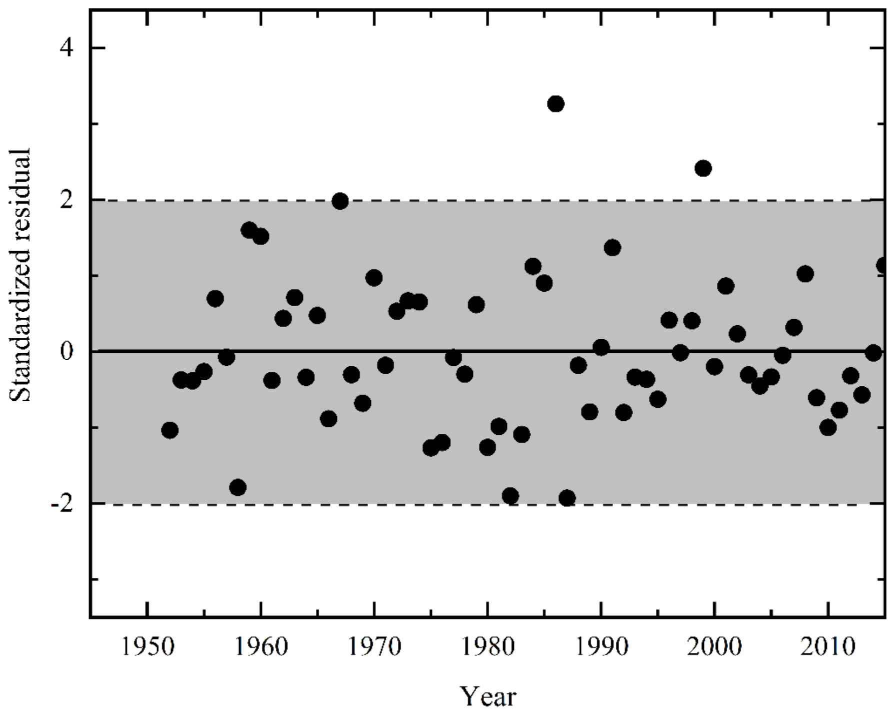

Fig. S12. Inspection of the regression residuals of the δ^{13}C-based annual precipitation reconstruction. Standardized residuals between the P8–C7 precipitation record (i.e., the total precipitation from the previous August to the current July) and precipitation reconstructed via single regression analysis using tree-ring δ^{13}C. Points outside the grey shading indicate outliers.

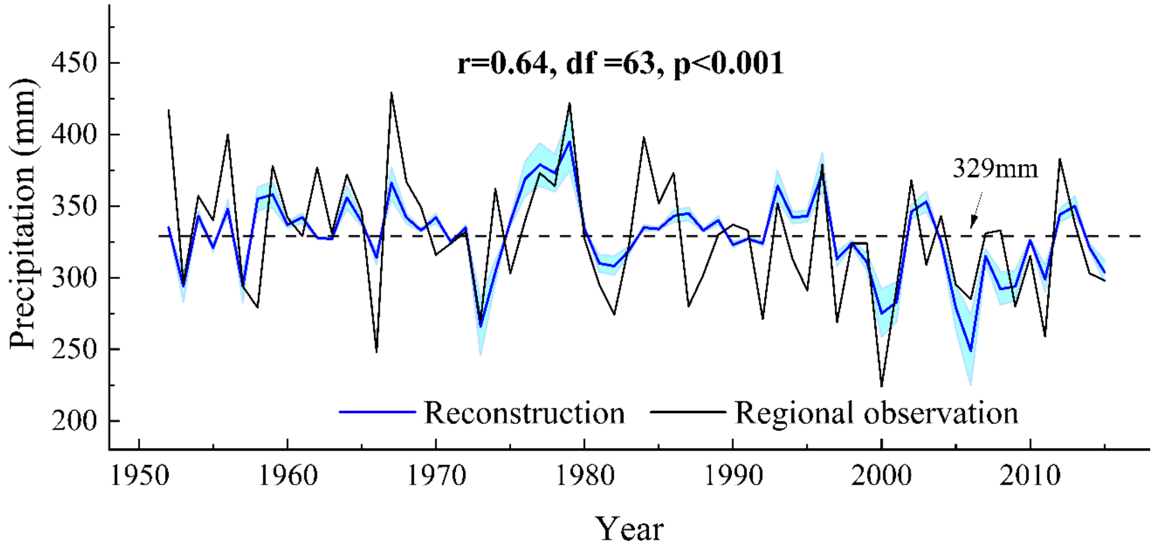

Fig. S13. Comparison between δ^{13}C-based regional reconstruction (blue line) and the mean regional P8-C7 precipitation (black line) record calculated from 39 stations during 1951-2015 CE. The shaded area indicates the 95% confidence level of predicted precipitation. The dashed line and its label are the average of instrumental precipitation records in 1951–2015 CE.

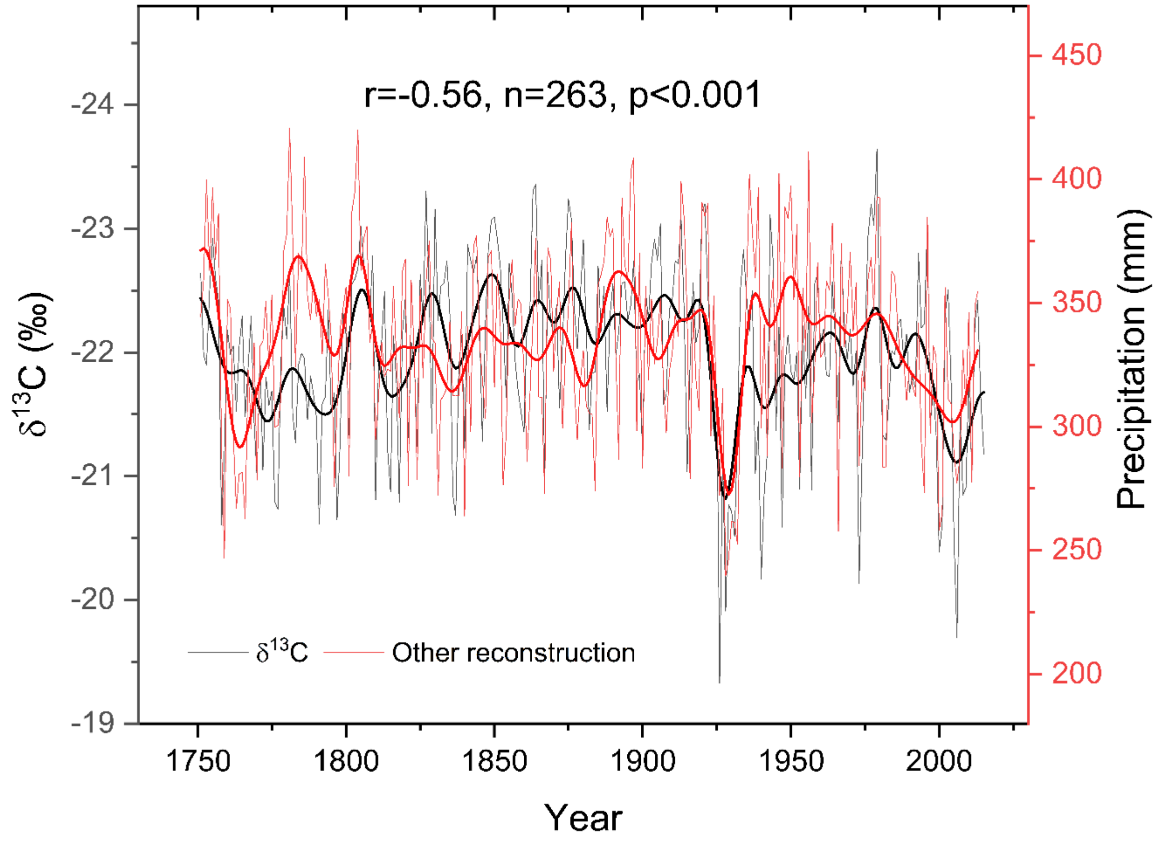

Fig. S14. Comparison between δ^{13}C chronology (black line) and the regional annual precipitation reconstruction (red line) at NASM. The bold line is smoothed with a 5-year FFT filter. The label is the correlation between δ^{13}C chronology and the regional precipitation reconstruction from ref. 21.

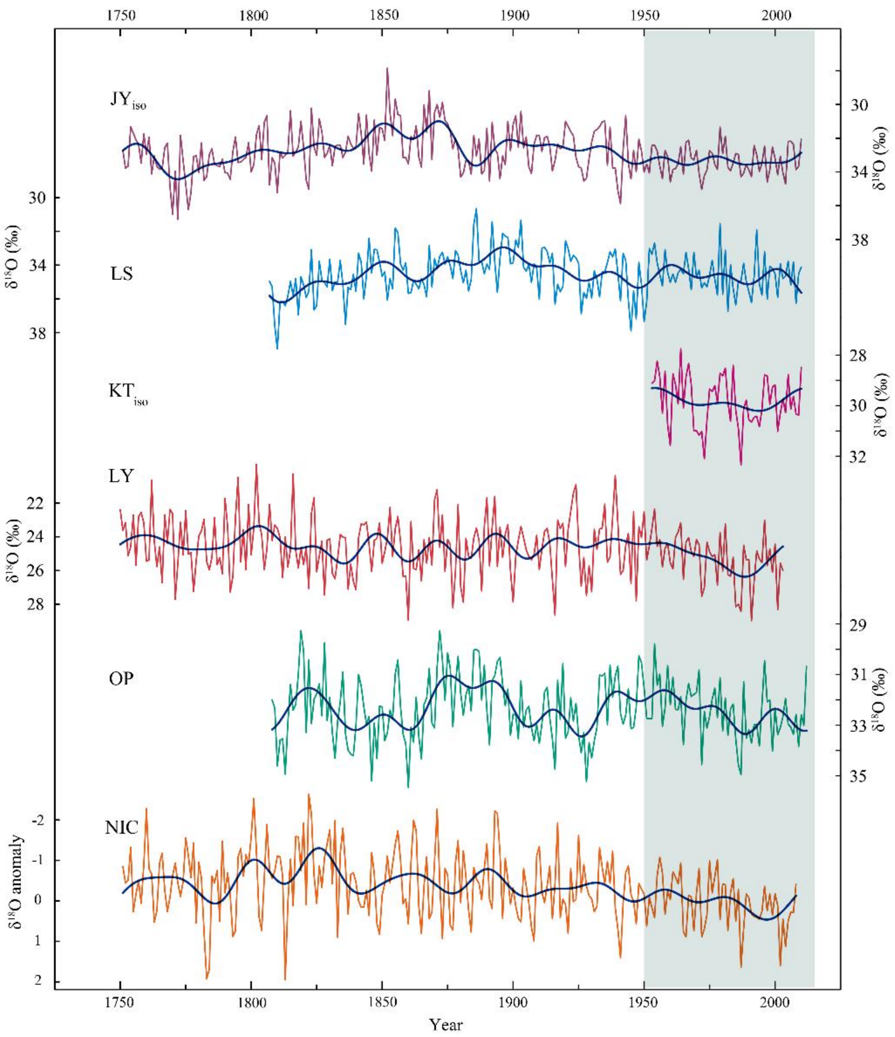

Fig. S15. Comparison between the new δ^{18}O series and other δ^{18}O records across the NASM region. The δ^{18}O record is from this study (JY_{iso}); KTiso represents C4–9 rH in North China (not published); information about the other tree-ring width and δ^{18}O records is listed in Table S5. The 50-year low-pass FFT smoothed curves (bold lines) emphasize decadal variations. All the tree-ring δ^{18}O records show a positive δ^{18}O trend during 1950-2010, indicative of decreasing moisture conditions, primarily linked to a weakening of the Asian summer monsoon.

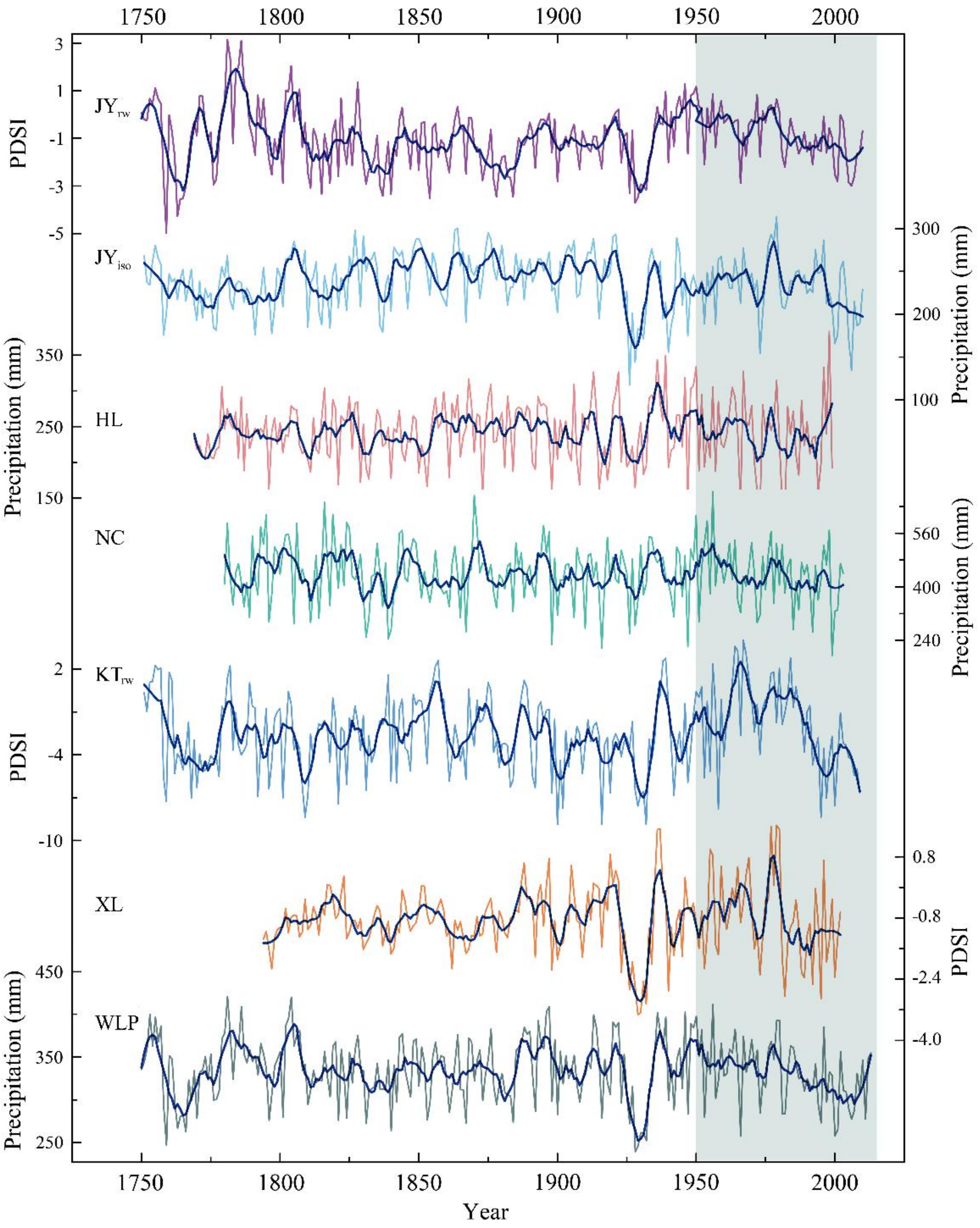

Fig. S16. Comparison of the new single-regression precipitation reconstruction with other hydroclimatic reconstructions from northern China during the period 1751–2010. JY_{rw}, P8-C7 precipitation and PDSI are based on ring widths at our study site (22). JY_{iso} and P8-C7 precipitation were reconstructed via single-regression analyses in this study; information on the other proxy records is listed in Table S5. The bold lines represent 20-year low-pass filter. All the hydroclimatic proxy records showed decreasing moisture conditions during 1950-2010, primarily linked to a weakening of the Asian summer monsoon.

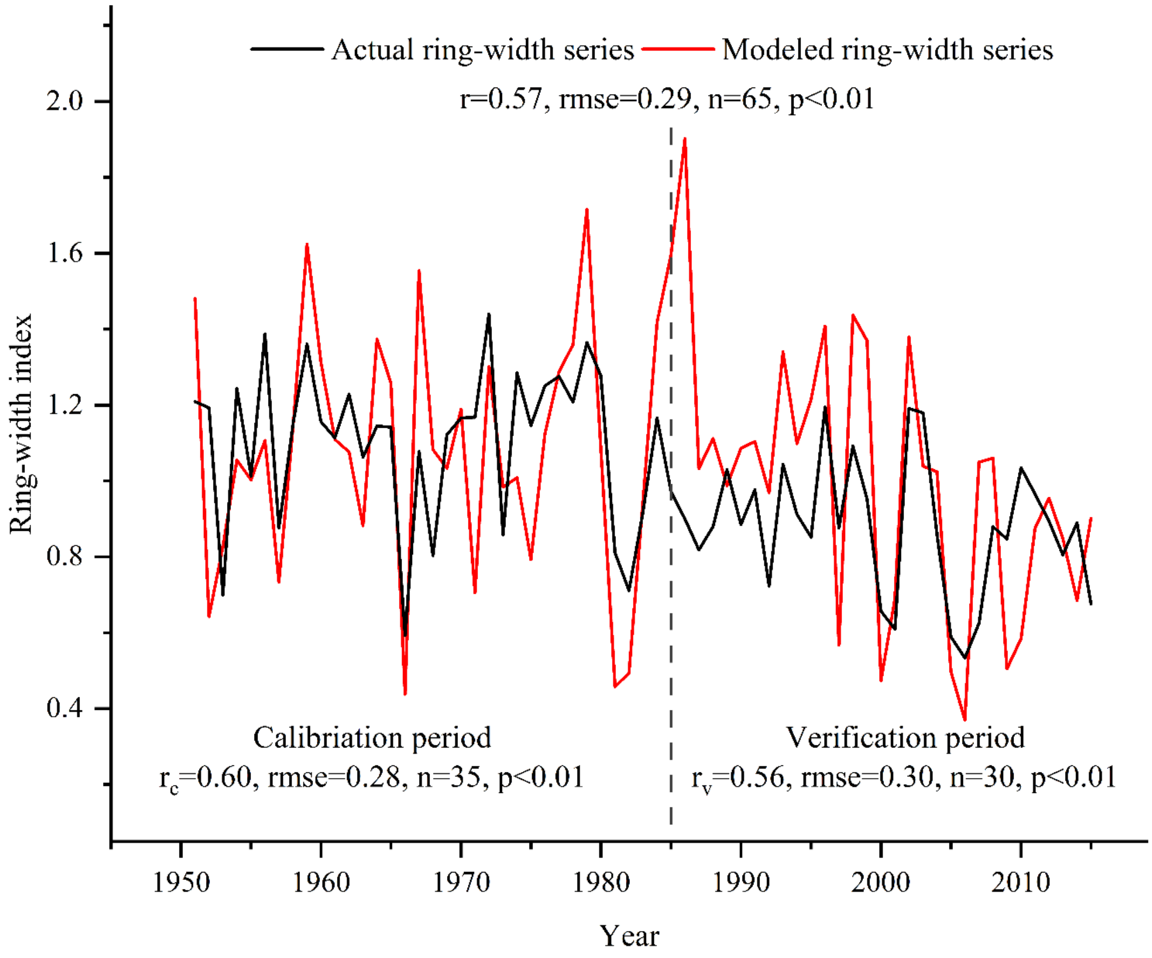

Fig. S17. Comparison between the actual and modeled ring-width chronologies. Comparison between STD (black line) and modeled (red line) from VS model during the calibration period (1951–1980) and verification period (1981–2015). r: Pearson correlation coefficient, rmse: root mean squared error.

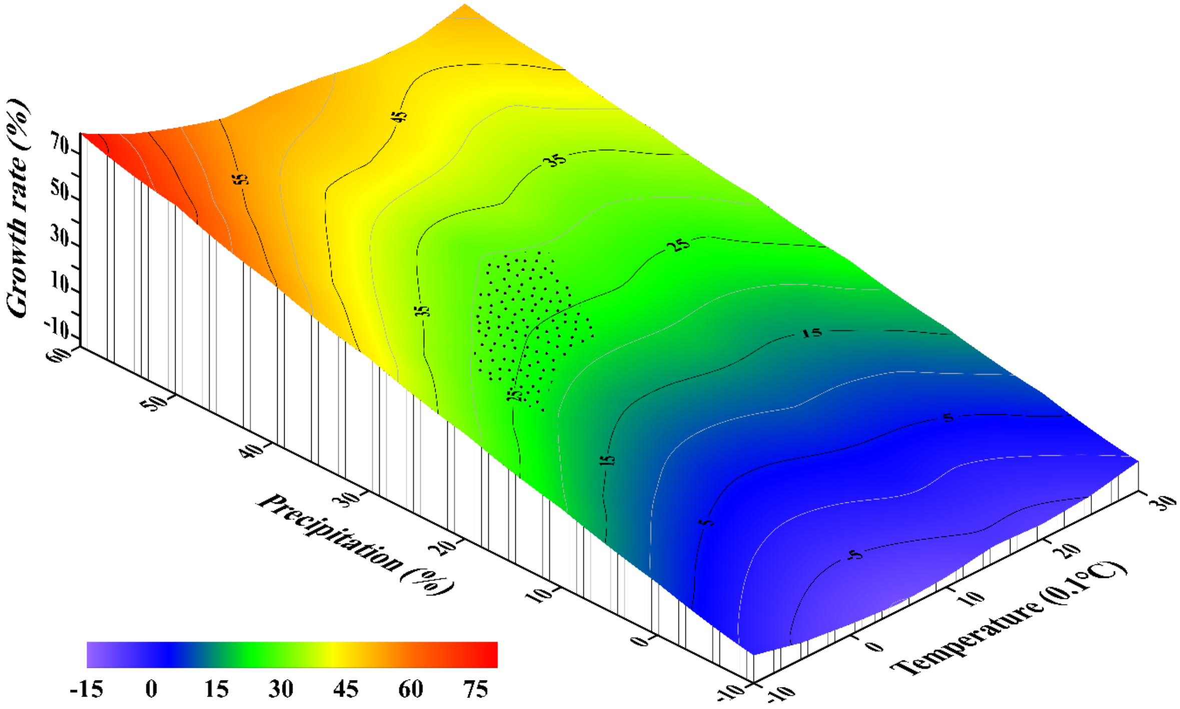

Fig. S18. Radial growth rates simulated by the VS model under different climate change scenarios. The climate conditions during 1951–2010 CE were used as the reference climate (i.e. zero indicates no change from current climate conditions). We performed sensitivity tests to assess the response of radial growth rates to different climate change scenarios by progressively modifying the climatic factors introduced into the model. Daily temperature and precipitation were modified by steps of 0.5 °C and 10%, within ranges of -1°C to +3°C and -10% to +60%, respectively. The shaded area with dots represents the range with precipitation increased by 16-34% under ± 0.5°C relative to 1951–2015 CE when the growth rates increase from 22% to 30% (Table S3). The surface was interpolated based on the unsmoothed growth rate series using the Kriging gridding method in Golden Surfer v21 (https://www.goldensoftware.com/products/surfer/).

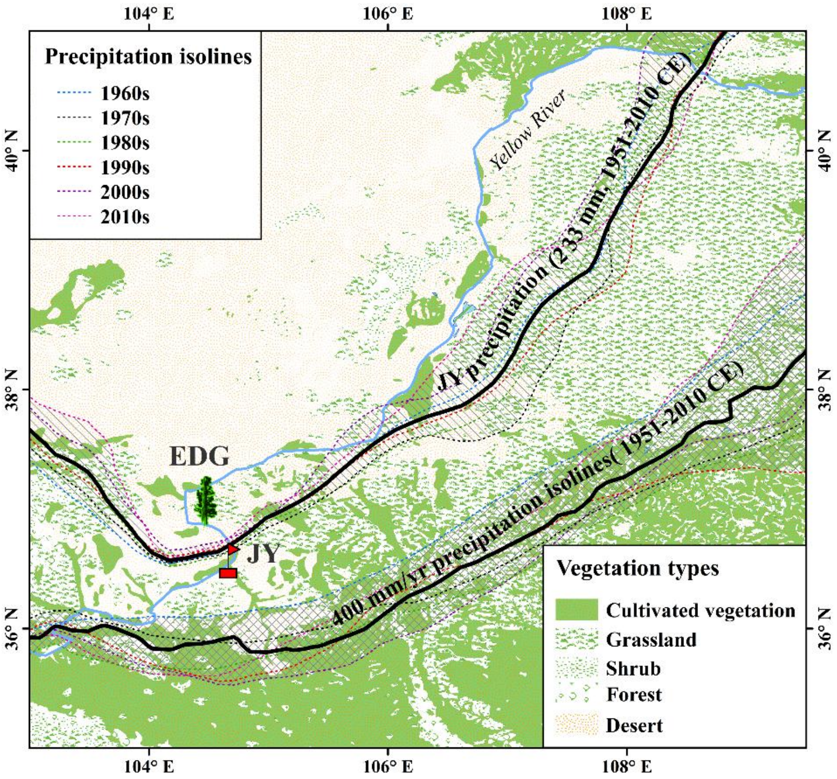

Fig. S19. Comparison of magnitude and direction of interdecadal variability of precipitation regimes between Jingyuan and the NASM region. Comparison of the interdecadal variation of the 233 mm/yr (based on the Jingyuan national meteorological station; JY, red flag) and 400 mm/yr precipitation isohyets. The green tree symbol indicates the sampling site EDG. The slash shaded area is the interdecadal scope of Jingyuan's isohyets during 1960s to 2010s (1960-2018 CE). The cross shaded area is the interdecadal scope of 400 mm/yr isohyets during 1960s-2010s. The isohyets are derived from interpolation of precipitation records from 613 national meteorological stations via the universal Kriging method. The vegetation records are taken from the vegetation map of the People's Republic of China (1:1000000) (23).

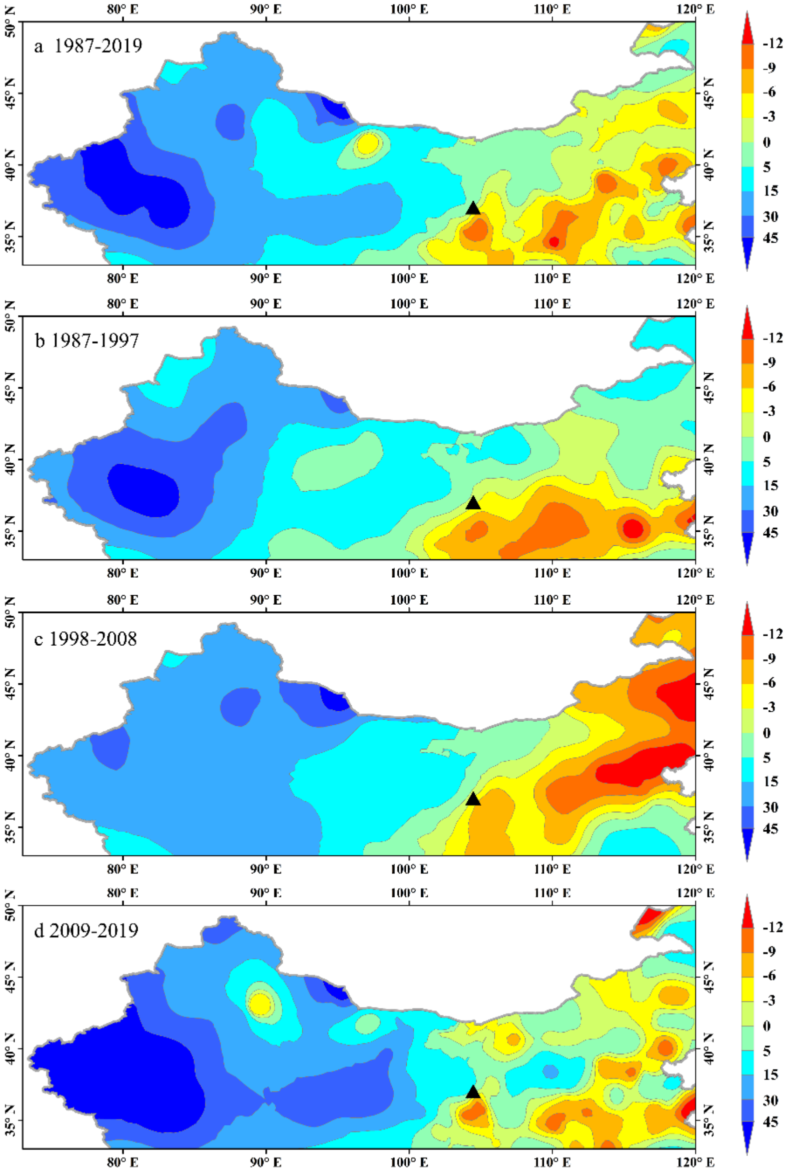

Fig. S20. Precipitation anomalies in NW China since 1987. Dynamic spatial distribution of annual precipitation anomalies (expressed as percentages) in northwestern China during 1987–2019. Spatial distribution of precipitation anomalies (relative to the 1957-1986 baseline) for time-means (a) 1987-2019, (b) 1987–1997, (c) 1998–2008 and (d) 2009–2019. The precipitation anomalies are interpolated from monthly instrumental records at 155 national weather stations via the co-kriging method, which considers the altitude of the stations. The red-to-blue shading indicates whether precipitation has decreased or increased in that area compared to 1957–1986. The black triangle indicates the LP sampling site.

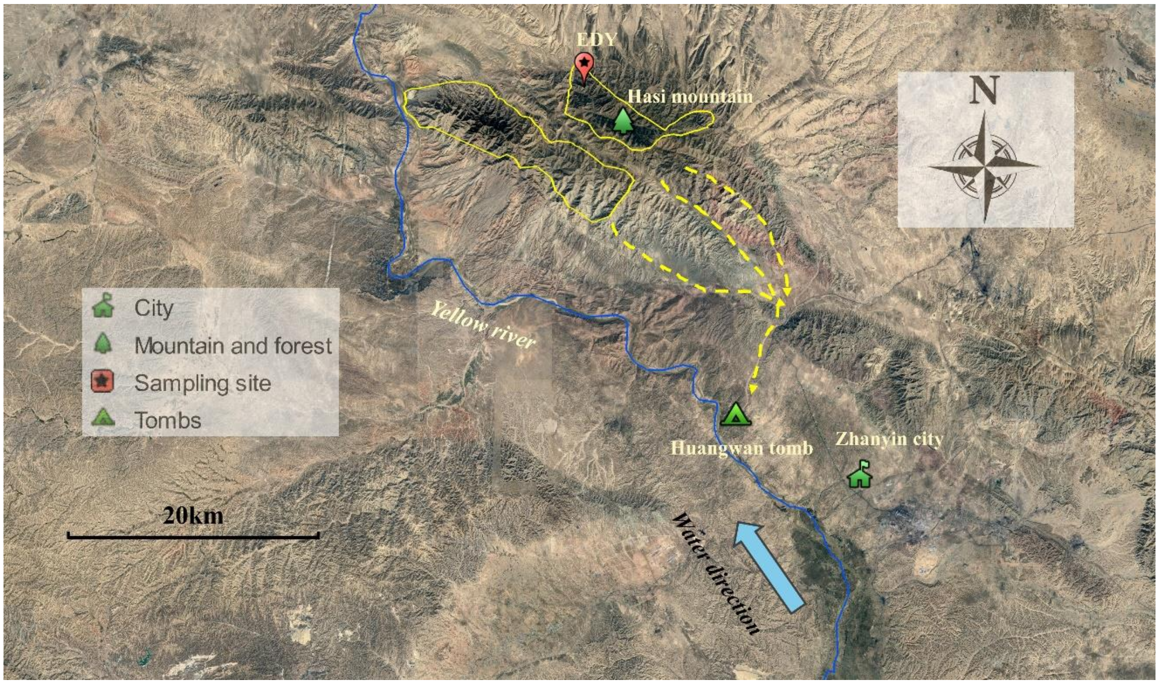

Fig. S21. Schematic diagram of the sources and transportation methods of wood from Huangwan tomb in Qin－Western Han dynasties. This map shows the location relationship of the main city (green house), main mountains and forests (green tree), Huangwan tomb (green triangle placemark) and sampling site (red placemark with black star) in this region at that time. The area enclosed by solid yellow lines is the forest reserve of Hasi at present. There are three possible routes (yellow dashed lines) to transport timbers for Huangwan tombs construction based on the documents and topographic maps.

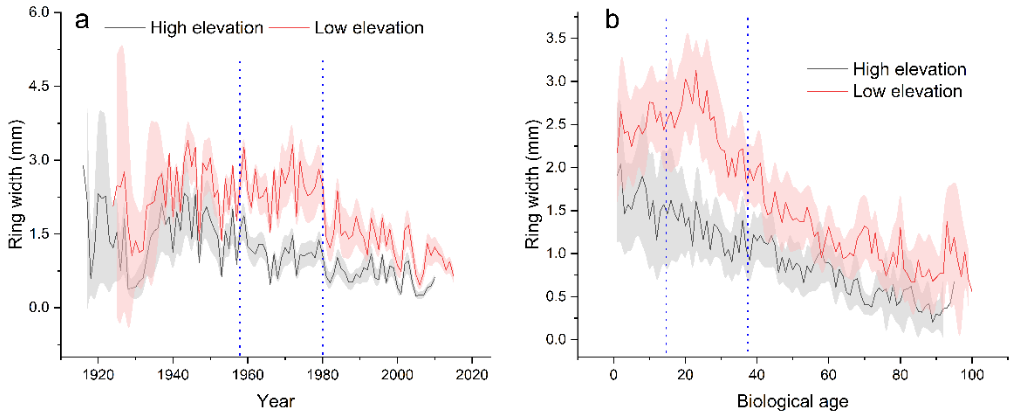

Fig. S22. Growth release of *Pinus tabulaeformis* at low altitudes on Hasi Mountain caused by artificial tending and felling (a) and its effect on radial growth (b). The black line (high elevation, 2382-2412m a.s.l.) and red line (low elevation, 2146-2296m a.s.l.) are the mean ring width from 12 trees and 13 trees with similar age (less than 100 years) respectively. The shaded areas indicate the 95% confidence ranges. The onset of large-scale artificial tending in 1958 triggered a more substantial growth release at lower elevations. This effect was further amplified by intensive felling activities during the period from 1965 to 1972.The growth release exhibited distinct patterns at different altitudes, with more pronounced effects at lower elevations (especially during the period highlighted by blue dashed lines).

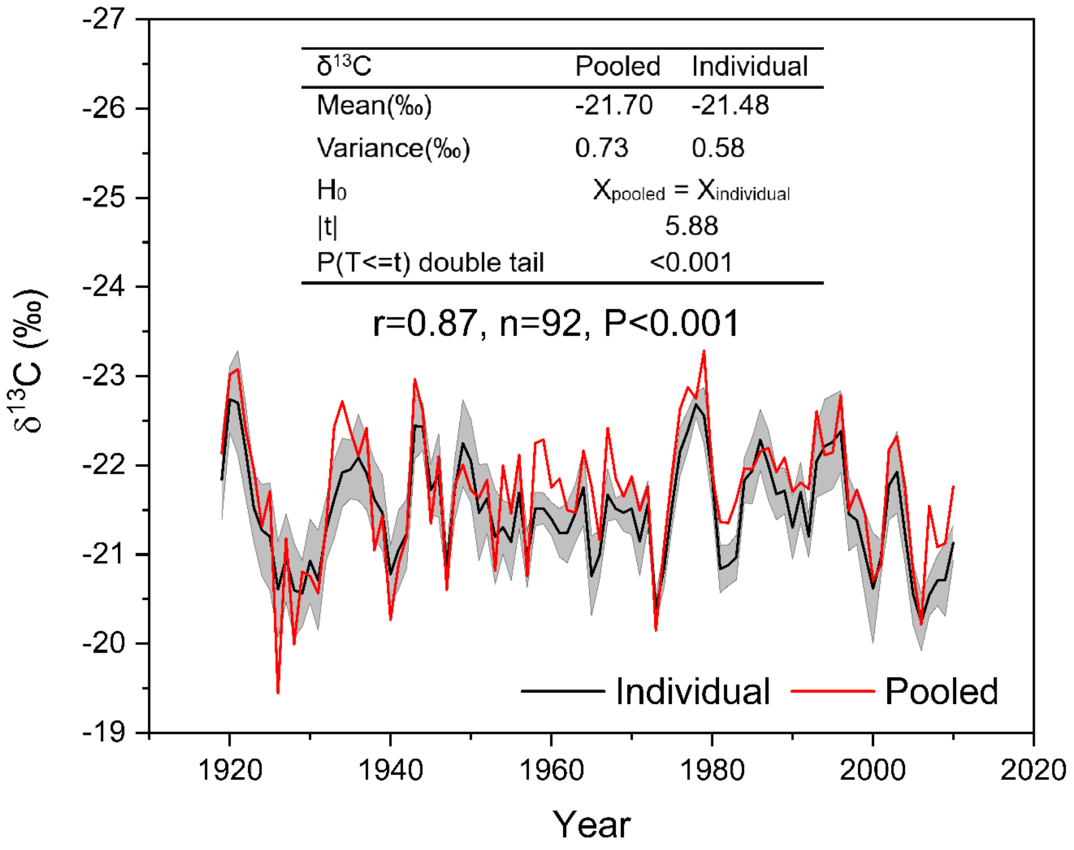

Fig. S23. Inter-laboratory comparison of δ^{13}C series analyzed in the laboratories in Friedrich-Alexander-Universität Erlangen-Nürnberg, Germany (with 10 trees pooling method, red line) and in Swansea University, United Kingdom (mean series of another 9 trees measured individually, black line) during 1919 – 2010CE. The grey shaded area represents the 95% confidence intervals for individual series. The inset table illustrates significance test results for the difference in δ^{13}C measurements between the laboratories at a level of α=0.05. Significant differences in mean value of δ^{13}C were detected. The δ^{13}C measurement via the individual tree method is from ref. 33.

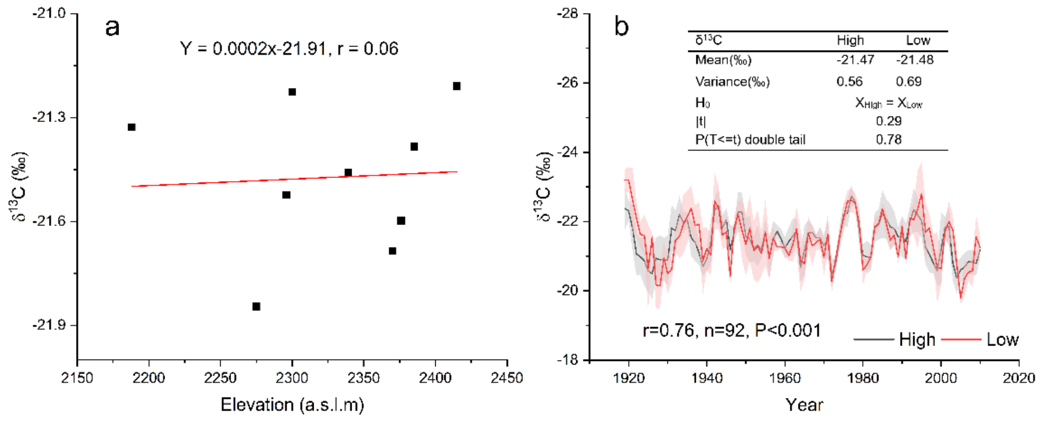

Fig. S24. Relationship between δ^{13}C series and sampling-tree elevation during 1919 − 2010 CE (a) and comparison of mean δ^{13}C series from high elevation (5 trees, black line) and low elevation (4 trees, red line) during 1919 − 2010CE (b). The grey shaded area represents the 95% confidence intervals for individual series. The inserted table illustrates significance test results for the difference in δ^{13}C measurements between the laboratories at a level of α=0.05. There is no significant trend between the elevation and the mean value of δ^{13}C. No significant differences in δ^{13}C measurements from high and low elevation were detected.

Table S1. Overview of wood samples used for isotopic analysis. Details of single samples of Chinese pine of living pine trees (LP) and archeological wood (AW) used for $\delta^{13}C$ and $\delta^{18}O$ measurement in this study.

Type	Code	Start	End	Length (years)	Elevation (m. a.s.l.)
Living Pine	EDY01A	1747	2010	264	2338
	EDY02B	1723	2010	288	2342
	EDY15A	1734	2010	277	2423
	EDY22B	1803	2002	200	2456
	EDY24B	1892	2010	119	2440
	EDY28A	1777	2010	234	2430
	EDY30A	1767	2010	244	2426
	EDY32A	1751	2010	260	2412
	EDY37A	1699	2010	312	2402
Archeological wood	JGP023B	-274	-89	186	-
	JGP032A	-336	-77	260	-
	JGP036A	-244	-55	190	-
	JGP038A	-269	-39	231	-
	JGP040A	-270	-104	167	-
	JGP044A	-257	-38	220	-
	JGP045A	-259	-78	182	-
	JGP047A	-282	-69	214	-
	JGP052A	-270	-88	183	-
	JGP056A	-262	-77	186	-

Table S2. Tree-ring width statistics between the LP and AW samples. Results of variance homogeneity analysis and mean test (α=0.05) of mean tree-ring width (mTRW), mean sensitivity (MS), first-order autocorrelation (AC1) and standard deviation (SD) between AW and LP samples in their corresponding credible periods (270–77 BCE and 1751–2010 CE, respectively). The ring-width unit is mm, and the cambial age unit is biological years. N denotes the number of trees. The null hypothesis that the LP and AW means are the same is rejected for all four statistics.

Item	mTRW		MS		AC1		SD	
	AW	LP	AW	LP	AW	LP	AW	LP
Mean	1.34	1.08	0.20	0.41	0.77	0.69	0.58	0.70
Variance	0.13	0.25	0.00	0.01	0.03	0.01	0.03	0.08
N	26	155	26	155	26	155	26	155
F	0.50		0.18		2.62		0.40	
P(F<=f)	0.02		0.00		0.00		0.00	
F single tail critical	0.57		0.57		1.58		0.57	
H_0	$\bar{X}_{AW}=\bar{X}_{LP}$		$\bar{X}_{AW}=\bar{X}_{LP}$		$\bar{X}_{AW}=\bar{X}_{LP}$		$\bar{X}_{AW}=\bar{X}_{LP}$	
|t|	3.30		23.08		2.11		2.76	
P(T<=t) single tail	0.00		0.00		0.04		0.01	
t single tail critical	2.02		1.99		2.05		2.01	

Table S3. Demonstration that the wider AW ring widths compared with LP ring widths are not dependent on biological age of the rings. Mean ring width (mTRW) ±1.96 standard error (the 95% confidence level) of LP and AW samples, and their difference (AW–LP)/LP as a percentage, for different biological age classes. Mean sample depth was no fewer than five trees for both LP and AW samples and for all ages classes. Considering the possible altitude effects between AW and LP samples, and the uncertainty caused by the recent growth release of low-altitude trees, we regard the growth rates at each growth stage from 1 to 220 years (sample depth⩾5 trees and relatively stable) as a reasonable range.

Biological age (year)	LP mTRW (mm)	AW mTRW (mm)	mTRW difference (%)
1–50	1.53±0.16	1.90±0.27	24*
1–100	1.27±0.13	1.55±0.22	22*
1–150	1.06±0.12	1.33±0.20	26*
1–200	0.93±0.11	1.19±0.19	28*
151–220	0.56±0.09	0.73±0.11	30*

* denotes that the differences in mTRW are significant at a level of α=0.05.

Table S4. Demonstration that the mean tree-ring isotopic variables are significantly different between the LP and AW samples. Results of the variance homogeneity analysis and mean test (α=0.05) of $\delta^{13}C$ and $\delta^{18}O$ chronologies from AW and LP samples in their corresponding credible periods (270–77 BCE and 1751–2015 CE) and instrumental period (1951–2015 CE, LP').

Item	$\delta^{13}C$			$\delta^{18}O$		
	AW	LP	LP'	AW	LP	LP'
Mean	-22.70	-21.98	-21.86	31.77	32.83	33.34
Variance	0.35	0.53	0.57	1.91	1.69	0.67
N	194	265	65	194	260	60
F		0.65	0.64		1.13	2.87
P(F<=f)		0.00	0.01		0.18	0.00
F single tail critical		0.82	0.72		1.25	1.44
H_0		$\bar{X}_{AW}=\bar{X}_{LP}$	$\bar{X}_{AW}=\bar{X}_{LP'}$		$\bar{X}_{AW}=\bar{X}_{LP}$	$\bar{X}_{AW}=\bar{X}_{LP'}$
|t|		11.73	8.47		8.37	10.87
P(T<=t) single tail		0.00	0.00		0.00	0.00
t single tail critical		1.65	1.66		1.65	1.65

Table S5. Details of other tree-ring-based hydroclimate proxy records used in this study.

Site No.	Full site name (Abbr.)	Lat.(°N)	Long.(°E)	Tree species	Proxy type	Notes	References
1	Longshou Mountain (LS)	39.05	100.07	*Picea crassifolia*	Tree-ring $\delta^{18}O$	The $\delta^{18}O$ chronology (1806-2011 CE) is significantly negatively correlated ($r = -0.740$, $p < 0.0001$) with summer (June-August) relative humidity at nearby climatic stations, and has a significant ($p < 0.01$) correlation with several EASM-related hydroclimate reconstructions over north and north-central China.	Li et al. (24)
2	The western Loess Plateau (WLP)	33.8−40.5	100−107	*Pinus tabulaeformis Carr, Picea crassifolia Kom., Picea purpurea Mast*	Tree-ring width	A composite (1566-2013 CE) of 10 tree ring-width chronologies from the west-central margin of the Asian summer monsoon region. This chronology correlates significantly ($r = -0.77$, $p < 0.0001$) with regional precipitation from July of previous year to June of current year over the calibration period from 1952 to 2013.	Liu et al. (21)
3	The Helan Mountain (HL)	39.08	106	*Pinus tabulaeformis*	Tree-ring width	The ring-width chronology (1788-1999 CE) is significantly correlated ($r = -0.68$, $p < 0.0001$) with March-July Palmer drought severity index (PDSI) at the nearest grid point.	Li et al. (25)
4	Kongtong Mountain (KT)	35.9	106.85	*Pinus tabulaeformis, Pinus armandidi*	Tree-ring width	The ring-width chronology (1615-2009 CE) is significantly correlated ($r = -0.84$, $p < 0.0001$) with May-July Palmer drought severity index (PDSI) at the nearest grid point.	Fang et al. (26)
5	Xinglong Mountain (XL)	35.5-36	103.8−104.1	*Picea wilsonii*	Tree-ring width	The ring-width chronology (1794-2003 CE) is significantly correlated ($r = 0.63$, $p < 0.0001$) with Palmer drought severity index (PDSI) from the previous August to current July at the nearest grid point.	Fang et al. (27)
6	The Ordos Plateau (OP)	39.37	110.75	*Pinus tabulaeformis Carr.*	Tree-ring $\delta^{18}O$	The $\delta^{18}O$ record (1808-2012 CE) significantly correlates ($r = -0.65$, $p < 0.0001$) with precipitation from June to August at a nearby station, and is significantly correlated with four Asian Summer Monsoon Indices defined by various methods ($p < 0.05$).	Liu et al. (28)

7	Luya Mountain (LY)	38.73	111.8	*Larix principis-rupprechtii*	Tree-ring $\delta^{18}O$	The $\delta^{18}O$ record (1779-2003 CE) is significantly negatively correlated ($r = -0.54$, $p < 0.0001$) with the previous August to current July precipitation at nearby climatic stations, and in combination with the ring-width chronology is significantly correlated with All Indian Precipitation records ($r = 0.32$, $n = 132$, $p < 0.001$).	Li et al. (29)
8	Two sites in Shanxi Province, North China (NC)	38.73<br>38.83	111.8<br>112.08	*Pinus tabulaeformis, Larix principis-rupprechtii*	Tree-ring width<br>Tree-ring $\delta^{18}O$	A regional precipitation record (1779-2003 CE) combining tree-ring widths and stable oxygen isotopes from two different tree species at two sites in North China. This composite is significantly correlated ($r = 0.72$, $p < 0.0001$) with the previous August to current July precipitation from nearby climatic stations, and significantly correlates with All Indian Precipitation records ($r = 0.32$, $n = 132$, $p < 0.001$).	Li et al. (29)
9	The northern Indian sub-continent (NIC)	28.0−32.22	77.22−90.0	*Cedrus deodara, Abies spectabilis, Abies pindrow, Larix griffithii*	Tree-ring $\delta^{18}O$	A regional composite (1743-2008 CE) of tree-ring oxygen isotope chronologies from five sites over the northern Indian subcontinent. The regional $\delta^{18}O$ record is significantly correlated with all India rainfall ($r=-0.5$, $p < 0.001$, $n=138$).	Xu et al. (30)

Table S6. Correlation analysis between different δ^{18}O records in the ASM region during the common period 1808-2003 CE. Detailed information is listed in Table S5. Where two values are given, they denote correlation coefficients with the original series and after 20-year low-pass filtering. KT_{iso} represents rH in C7-9 (r=-0.56, n=58, p<0.01, 1953–2010 CE) in northern China (this study), without the second value. Bold values are significant at the α=0.01 level; gray values are insignificant at the α=0.05 level.

	JY_{iso}	LS	KT_{iso}	LY	OP	NIC
JY_{iso}	**1.00**					
LS	**0.39/0.29**	**1.00**				
KT_{iso}	**0.57**	0.29	**1.00**			
LY	**0.37/0.36**	**0.32/0.22**	**0.57**	**1.00**		
OP	**0.26**/-0.02	**0.35/0.34**	**0.45**	**0.45/0.40**	**1.00**	
NIC	**0.21/0.43**	-0.04/-0.06	**0.43**	**0.20/0.33**	**0.28/0.25**	**1.00**

Table S7. Regression model of annual precipitation on tree-ring $\delta^{13}C$. Model summary of reconstructed P8-C7 (previous August to current July) precipitation from Jingyuan meteorological station using single-regression analysis based on $\delta^{13}C$ during 1951–2015.

Model [a]	R	R^2	R_{adj}^2	Std. Error of the Estimate	Durbin-Watson [b]
1	.57	0.32	0.31	48.92	1.96

[a] Predictors: (constant), $\delta^{13}C$; dependent variable: P8-C7 precipitation. [b] A value close to 2 indicates that the residual term is not autocorrelated.

Table S8. Analysis of variance (ANOVA) for tree-ring $\delta^{13}C$ regression equation for P8-C7 precipitation.

Model	Sum of squares	df	Mean square	F	Sig.
Regression	70100.27	1	70100.27	29.30	.000
Residual	148326.71	62	2392.95		
Total	2218462.98	63			

Table S9. Correlation analysis between tree-ring-based hydroclimatic reconstructions in northern China during the common period 1794–1999 CE. Detailed information on each series is listed in Table S5. The two values denote correlation coefficients with the original series and after 20-year low-pass filtering. Bold values are significant at the α=0.01 level; gray values are insignificant at the α=0.05 level.

	JY_{rw}	JY_{iso}	HL	NC	KT_{rw}	XL	WLP
JY_{rw}	**1.00**						
JY_{iso}	**0.47**/0.06	**1.00**					
HL	**0.48/0.21**	**0.40**/-0.09	**1.00**				
NC	**0.40/0.37**	**0.31**/0.01	**0.46/0.32**	**1.00**			
KT_{rw}	**0.43/0.41**	**0.26/0.32**	**0.39**/0.09	**0.27**/0.08	**1.00**		
XL	**0.45/0.38**	**0.44/0.41**	**0.34/0.24**	**0.21/0.22**	**0.45/0.57**	**1.00**	
WLP	**0.80/0.79**	**0.56/0.48**	**0.67/0.28**	**0.44/0.38**	**0.64/0.43**	**0.63/0.68**	**1.00**

Table S10. Parameters and values of the VS tree-growth model used in this study.

Parameter	Description	Value
Tmin	Minimum temperature for tree growth (°C)	6.000
Topt1	Lower end of range of optimal temperatures (°C)	13.000
Topt2	Upper end of range of optimal temperatures (°C)	19.000
Tmax	Maximum temperature for tree growth (°C)	29.000
Wmin	Minimum soil moisture for tree growth, relative to saturated soil (v/vs)	0.025
Wopt1	Lower end of range of optimal soil moistures (v/vs)	0.125
Wopt2	Upper end of range of optimal soil moisture (v/vs)	0.250
Wmax	Maximum soil moisture for tree growth (v/vs)	0.300
W0	Initial soil moisture (v/vs)	0.075
Ww	Minimum soil moisture (wilting point, v/vs)	0.050
Tbeg	Sum of temperature to start growth (°C)	135.000
Pmax	Maximum daily precipitation for saturated soil (mm/day)	20.000
C1	Fraction of precipitation penetrating soil (not caught by crown)	0.190
C2	First coefficient for calculation of transpiration (mm/day)	0.120
C3	Second coefficient for calculation of transpiration (mm/day)	0.115
Vcr	Critical growth rate	0.080
Ir	Root depth(mm)	1000.000
Cd	the rate of drainage water from soil	0.001
Cs	Coefficient for solar modification	0.960

Table S11. The precipitation reconstruction series in this study captures various types of historical extreme climate events and related social-economic activities that were recorded in historical documents. The pointer years are defined as years in our precipitation reconstruction that fall outside the range of +/- 1.5 standard deviations during 270-77 BCE. The mean precipitation is 271mm with 27mm standard deviations during this period. To eliminate the confusion caused by the year zero in this study, we uniformly subtract 1 from our dates and compare the adjusted date with historical documents. The reconstruction in this study represents precipitation from previous August to current July. There are 8 out of 22 events which are also recorded by historical documents.

Pointer year (BCE)	Reconstruction (mm)	Documented climate events	Extent of climate events	Source	Reference
228	227	Heavy famine	Shanggu; Guanzhong area	“Shiji·Qinshihuangbenji", ref.36	ref. 35, 37
190	230	Severe drought in the summer, which caused the water levels of rivers to drop and even some streams to dry up.	Gansu; Shanxi	“Hanshu·Wuxingzhi", ref.36	ref. 35, 37, 38
158	220	The entire region experienced severe drought in the spring, which intensified in April and was accompanied by a locust plague. Government aids: reducing taxes, reducing tributes, resting and recuperating, reducing expenditures, distributing grain for disaster relief, and buying/selling official positions. Borer plague in August.	Gansu; Hebei xuanhua, weizhou; Guanzhong area	“Shiji·Benji”,“Hanshu·Benji”,“Xunji”,“Hanshu·Wuxingzhi”,“Hanshu·Wendiji”, ref.36	ref. 34, 35, 37
146	212	Severe drought in autumn of 147BCE; A plague of locusts in March and summer of 146 BCE	Gansu; Guanzhong	“Shiji·Xiaojingbenji",“ Hanshu·Wuxingzhi", ref.36	ref. 35, 37
128	209	Severe drought in the summer of 129BCE, which caused locust plague, crop failure and famine.	Gansu; Henan taikang; Shanxi; Hebei weixian	"Zizhi Tongjian·Hanji", “Hanshu·Wuxingzhi",	ref. 35, 37

				“Hanshu·Wudiji”, ref.36	
86	311	Heavy rains lasted during July and October, and the Wei Bridge was completely destroyed.	Guanzhong area	“Hanshu·Zhaodiji”,“Hanshu·Wuxingzhi”	ref. 35
85	318	Heavy rains lasted from July to October of 86 BCE.	Guanzhong area	“Hanshu·Zhaodiji”,“Hanshu·Wuxingzhi”	ref. 35, 38
81	228	Drought in Summer, heavy fog, no fire allowed.	Guanzhong area	“Hanshu·Zhaodiji”, ref.36	ref. 35, 37, 38

*Guanzhong area is in the alluvial plain of the lower reaches of the Wei River, with an area of 56,000 square kilometers and was an important agricultural production area during the Qin–Western Han dynasties. Shanggu is located in Zhangjiakou city of Hebei province. Events with not clear extent were regarded as Guanzhong area taking into account that the capital at that time locate at this area.

SI References

1. F. Xu, *Anatomical figures for wood identification* (Chemical Industry Press, Beijing, 2008).
2. Y. Q. Du, F. T. Chen, On the Relationship between the Zhanyin City and the Huangwan Tombs. *The Silk Road*, 21-26 (2015).
3. Baiyin City Local Chronicles Compilation Committee, Baiyin City Annals (Zhonghua Book Company, 1999).
4. Z. J. Wang, A historic investigation of climate change in the Qin and Han Dynasties. Historical Research 2, 3-19 (1995).
5. F. Wang, Research on Forest Ecological Civilization in the Pre-Qin and Han Dynasties (China Social Sciences Press, 2015).
6. Gansu Provincial Local History Compilation Committee, Gansu Provincial Forestry Compilation Committee. Gansu Provincial Annals Volume 12: Forestry Annals (Gansu People's Publishing House, 1999).
7. F. Rinntech, *Time series analysis and presentation for dendrochronology and related applications* (User Reference, Rinntech, Heidelberg, 2005).
8. R. L. Holmes, *Program COFECHA user's manual* (Laboratory of Tree-Ring Research, The University of Arizona, Tucson, 1983).
9. M. Q. Li, X. M. Shao, Z. Y. Yin, X. G. Xu, Tree-Ring Dating of the Reshui-1 Tomb in Dulan County, Qinghai Province, North-West China. *PLoS One* **10**, e0133438 (2015).
10. B. Yang *et al.*, A 3,500-year tree-ring record of annual precipitation on the northeastern Tibetan Plateau. *Proc. Natl. Acad. Sci. U.S.A.* **111**, 2903-2908 (2014).
11. I. Hajdas, Radiocarbon dating and its applications in Quaternary studies. *Eiszeitalter und Gegenwart Quaternary Science Journal* **57**, 2-24 (2008).
12. P. J. Reimer *et al.*, IntCal13 and Marine13 Radiocarbon Age Calibration Curves 0–50,000 Years cal BP. *Radiocarbon* **55**, 1869-1887 (2013).
13. A. S. Talma, J. C. Vogel, A Simplified Approach to Calibrating 14C Dates. *Radiocarbon* **35**, 317-322 (1993).
14. R. J. Francey, G. D. Farquhar, An explanation of $^{13}C/^{12}C$ variations in tree rings. *Nature* **297**, 28-31 (1982).
15. D. McCarroll, N. J. Loader, Stable isotopes in tree rings. *Quaternary Sci. Rev.* **23**, 771-801 (2004).
16. A. Gessler *et al.*, Stable isotopes in tree rings: towards a mechanistic understanding of isotope fractionation and mixing processes from the leaves to the wood. *Tree Physiol.* **34**, 796-818 (2014).
17. J. S. Roden, G. G. Lin, J. R. Ehleringer, A mechanistic model for interpretation of hydrogen and oxygen isotope ratios in tree-ring cellulose. *Geochim. Cosmochim. Acta* **64**, 21-35 (2000).
18. A. Gessler, K. Treydte, The fate and age of carbon–insights into the storage and remobilization dynamics in trees. *New Phytol.* **209**, 1338-1340 (2016).
19. K. Treydte *et al.*, Seasonal transfer of oxygen isotopes from precipitation and soil to the tree ring: source water versus needle water enrichment. *New Phytol.* **202**, 772-783 (2014).
20. M. He *et al.*, Recent advances in dendroclimatology in China. *Earth Sci. Rev.* **194**, 521-535 (2019).
21. Y. Liu *et al.*, Anthropogenic Aerosols Cause Recent Pronounced Weakening of Asian Summer Monsoon Relative to Last Four Centuries. *Geophys. Res. Lett.* **46**, 5469-5479 (2019).
22. S. Y. Kang, B. Yang, C. Qin, Recent tree-growth reduction in north central China as a combined result of a weakened monsoon and atmospheric oscillations. *Clim. Change* **115**, 519-536 (2012).
23. Chinese Vegetation Map Editorial Board of Chinese Academy of Sciences, *Vegetation map of the People's Republic of China (1: 1000000)* (Science Press, 2001).
24. Q. Li *et al.*, East Asian Summer Monsoon moisture sustains summer relative humidity in the southwestern Gobi Desert, China: evidence from $\delta^{18}O$ of tree rings. *Clim. Dynam.* **52**, 6321-6337 (2018).
25. J. B. Li, F. H. Chen, E. R. Cook, X. H. Gou, Y. X. Zhang, Drought reconstruction for North Central China from tree rings: the value of the Palmer drought severity index. *Int. J. Climatol.* **27**, 903-909 (2007).
26. K. Y. Fang *et al.*, Tree-ring based reconstruction of drought variability (1615–2009) in the Kongtong Mountain area, northern China. *Global Planet. Change* **80-81**, 190-197 (2012).

27. K. Y. Fang *et al.*, Drought variations in the eastern part of northwest China over the past two centuries: evidence from tree rings. *Clim. Res.* **38**, 129-135 (2009).
28. Y. Liu *et al.*, Asian Summer Monsoon - Related Relative Humidity Recorded by Tree Ring $\delta^{18}O$ During Last 205 Years. *J. Geophys. Res.: Atmos.* **124**, 9824-9838 (2019).
29. Q. Li *et al.*, The 225-year precipitation variability inferred from tree-ring records in Shanxi Province, the North China, and its teleconnection with Indian summer monsoon. *Global Planet. Change* **132**, 11-19 (2015).
30. C. X. Xu *et al.*, Decreasing Indian summer monsoon on the northern Indian sub-continent during the last 180 years: evidence from five tree-ring cellulose oxygen isotope chronologies. *Clim. Past* **14**, 653-664 (2018).
31. J. Barichivich, T. Osborn, I. Harris, G. van der Schrier, P. Jones, Monitoring global drought using the self-calibrating Palmer Drought Severity Index [in" State of the Climate in 2020" eds. Dunn RJH, Aldred F, Gobron N, Miller JB & Willett KM]. *Bull. Am. Meteorol. Soc.* **102**, S68-S70 (2021).
32. I. Harris, T. J. Osborn, P. Jones, D. Lister, Version 4 of the CRU TS monthly high-resolution gridded multivariate climate dataset. *Sci Data* **7**, 109 (2020).
33. S. Kang et al., Tree-Ring Stable Carbon Isotope as a Proxy for Hydroclimate Variations in Semi-Arid Regions of North-Central China. *Forests*, **13** (2022).
34. Q. S. Ge, *Climate change in Chinese dynasties* (Science Press, Beijing, 2011).
35. D. E. Zhang, *A Compendium of Chinese Meteorological Records of the Last 3,000 Years* (Jiangsu Education Press, Nanjing, 2013).
36. K. G.Wen, *China Meteorological Disasters Encyclopedia* (Meteorological Press, Beijing, 2008).
37. Y. H. Li, *A Record of Drought in China over the Past Three Thousand Years* (Meteorological Press, Beijing, 2021)
38. C. Y. Li, Climatic Change from the Late Warring States Period to the Xi Han. *Journal of Baoji University of Arts and Sciences (Social Sciences)*, **31** (2011).
